# Stream Dissolved Organic Matter in Permafrost Regions Shows Surprising Compositional Similarities but Negative Priming and Nutrient Effects

**DOI:** 10.1029/2020GB006719

**Published:** 2021-01-11

**Authors:** Ethan Wologo, Sarah Shakil, Scott Zolkos, Sadie Textor, Stephanie Ewing, Jane Klassen, Robert G. M. Spencer, David C. Podgorski, Suzanne E. Tank, Michelle A. Baker, Jonathan A. O'Donnell, Kimberly P. Wickland, Sydney S. W. Foks, Jay P. Zarnetske, Joseph Lee‐Cullin, Futing Liu, Yuanhe Yang, Pirkko Kortelainen, Jaana Kolehmainen, Joshua F. Dean, Jorien E. Vonk, Robert M. Holmes, Gilles Pinay, Michaela M. Powell, Jansen Howe, Rebecca J. Frei, Samuel P. Bratsman, Benjamin W. Abbott

**Affiliations:** ^1^ Department of Land Resources and Environmental Sciences Montana State University Bozeman MT USA; ^2^ Department of Biological Sciences University of Alberta Edmonton Alberta Canada; ^3^ Woods Hole Research Center Woods Hole MA USA; ^4^ Department of Earth, Ocean and Atmospheric Science and National High Magnetic Field Laboratory Geochemistry Group Florida State University Tallahassee FL USA; ^5^ Department of Biology and Ecology Center Utah State University Logan UT USA; ^6^ Arctic Network National Parks Service Anchorage AK USA; ^7^ Water Resources Mission Area USGS Boulder CO USA; ^8^ Department of Earth and Environmental Sciences Michigan State University East Lansing MI USA; ^9^ State Key Laboratory of Vegetation and Environmental Change, Institute of Botany Chinese Academy of Sciences Beijing China; ^10^ Finnish Environment Institute SYKE Joensuu Finland; ^11^ Department of Earth Sciences Vrije Universiteit Amsterdam Amsterdam Netherlands; ^12^ School of Environmental Sciences University of Liverpool Liverpool UK; ^13^ Environnement‐Ville‐Société (UMR5600) ‐ Centre National de la Recherche Scientifique (CNRS) Lyon France; ^14^ Department of Plant and Wildlife Sciences Brigham Young University Provo UT USA; ^15^ Department of Renewable Resources University of Alberta Edmonton Alberta Canada

**Keywords:** permafrost, cryosphere and high‐latitude processes, thermokarst, rivers, carbon cycling, nutrients and nutrient cycling

## Abstract

Permafrost degradation is delivering bioavailable dissolved organic matter (DOM) and inorganic nutrients to surface water networks. While these permafrost subsidies represent a small portion of total fluvial DOM and nutrient fluxes, they could influence food webs and net ecosystem carbon balance via priming or nutrient effects that destabilize background DOM. We investigated how addition of biolabile carbon (acetate) and inorganic nutrients (nitrogen and phosphorus) affected DOM decomposition with 28‐day incubations. We incubated late‐summer stream water from 23 locations nested in seven northern or high‐altitude regions in Asia, Europe, and North America. DOM loss ranged from 3% to 52%, showing a variety of longitudinal patterns within stream networks. DOM optical properties varied widely, but DOM showed compositional similarity based on Fourier transform ion cyclotron resonance mass spectrometry (FT‐ICR MS) analysis. Addition of acetate and nutrients decreased bulk DOM mineralization (i.e., negative priming), with more negative effects on biodegradable DOM but neutral or positive effects on stable DOM. Unexpectedly, acetate and nutrients triggered breakdown of colored DOM (CDOM), with median decreases of 1.6% in the control and 22% in the amended treatment. Additionally, the uptake of added acetate was strongly limited by nutrient availability across sites. These findings suggest that biolabile DOM and nutrients released from degrading permafrost may decrease background DOM mineralization but alter stoichiometry and light conditions in receiving waterbodies. We conclude that priming and nutrient effects are coupled in northern aquatic ecosystems and that quantifying two‐way interactions between DOM properties and environmental conditions could resolve conflicting observations about the drivers of DOM in permafrost zone waterways.

## Key Points

- Dissolved organic matter (DOM) from diverse circumpolar regions showed compositional and optical commonalities
- Added acetate and nutrients suppressed background DOM degradation but accelerated colored DOM breakdown
- Disconnect between DOM composition and function indicates intrinsic and extrinsic conditions interact to regulate DOM dynamics

## 1 Introduction

Climate change is degrading permafrost at continental scales (Biskaborn et al., 2019; Jorgenson et al., 2018; Nitze et al., 2018; Olefeldt et al., 2016). Though the specific consequences of permafrost degradation depend on local conditions (Frey & McClelland, 2009; Littlefair et al., 2017; Tank et al., 2012, 2020; Toohey et al., 2016; Zolkos & Tank, 2020), the thawing of frozen material and associated changes in water flow are causing release of dissolved organic matter (DOM) and inorganic nutrients such as nitrogen (N) and phosphorous (P) to streams, lakes, and coastal zones (Kendrick et al., 2018; O'Donnell, Aiken, Swanson, et al., 2016; Tanski et al., 2017; Treat et al., 2016; Vonk, Tank, Bowden, et al., 2015; Wickland et al., 2018). Though permafrost‐derived DOM is often hundreds to tens of thousands of years old, it can be highly biolabile (Abbott et al., 2014; Drake et al., 2015; Ewing et al., 2015; Liu et al., 2019; Vonk et al., 2013) and photodegradable (Cory et al., 2013; Vonk, Tank, Bowden, et al., 2015), depending on source and permafrost type (Stubbins et al., 2016; Wickland et al., 2018). Consequently, some permafrost‐derived DOM can be rapidly mineralized in headwater streams (Drake et al., 2015; Mann et al., 2015; Spencer et al., 2015; Vonk, Tank, Mann, et al., 2015; Wickland et al., 2012). Waterways in the permafrost zone already transport globally relevant amounts of DOM and nutrients (Holmes et al., 2012; McClelland et al., 2014). For example, Arctic and Boreal surface waters receive ~100 Tg of dissolved organic carbon (DOC) each year from terrestrial ecosystems, a third of which (~35 Tg C yr^−1^) they deliver to the Arctic Ocean and surrounding seas (Abbott, Jones, et al., 2016; Kicklighter et al., 2013; McGuire et al., 2009). Radiocarbon measurements suggest that more than 80% of this DOC is modern—fixed since the 1950s (Qu et al., 2017; Raymond et al., 2007; Wild et al., 2019)—and even under extreme warming scenarios, DOM from degrading permafrost will likely remain a small proportion of total DOM flux (Abbott et al., 2015; Abbott, Jones, et al., 2016; Estop‐Aragonés et al., 2020; Laudon et al., 2012). However, when biolabile DOC (BDOC) and nutrients from permafrost mix with modern DOM, they could influence mineralization rates and alter the net ecosystem carbon balance of the permafrost zone (Abbott et al., 2014; Larouche et al., 2015; Textor et al., 2019), potentially resulting in greater CO_2_ efflux from permafrost ecosystems to the atmosphere.

**Figure 1:**
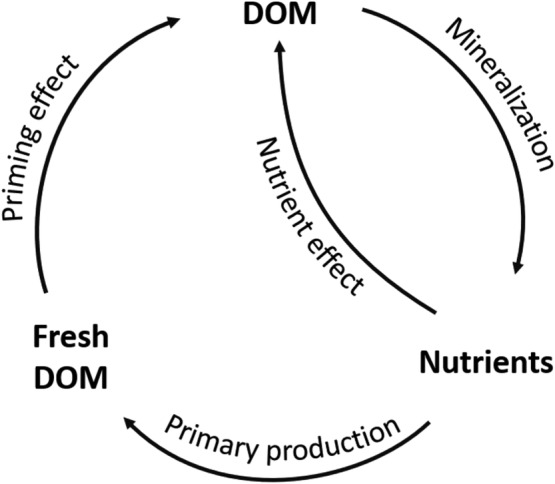
Schematic diagram of linkages between nutrient and priming effects in low‐nutrient ecosystems. Arrows represent processes that produce or influence the various pools of dissolved organic matter (DOM) and nutrients. For example, nutrients stimulate primary production, which creates fresh DOM. Subsequently, fresh DOM may exert a positive or negative priming effect on the overall DOM pool. In eutrophic ecosystems such as agricultural and urban environments, nutrient effects can be decoupled from priming by the addition of anthropogenic nutrients and removal of fresh DOM sources during harvesting.

Rates of DOM mineralization depend on the intrinsic properties of the DOM such as chemical composition as well as external conditions such as temperature, microbial community structure, and interactions with other elements (Abbott, Baranov, et al., 2016; Arnosti, 2004; Frei et al., 2020; Marín‐Spiotta et al., 2014; Nalven et al., 2020; Zarnetske et al., 2011). Even DOM that has low inherent reactivity because of its source or prior processing may undergo further mineralization and transformation when mixed with BDOC or inorganic nutrients (Bianchi, 2012; Guenet et al., 2010; Kuzyakov et al., 2000; Mutschlecner et al., 2018; Rosemond et al., 2015). The addition of BDOC and nutrients may relieve energy and stoichiometric limitations of decomposers, accelerating mineralization of stable organic matter in the short term and altering the type of organic matter exported in the long term (Chen et al., 2019; Guenet et al., 2010; Lynch et al., 2018; Mack et al., 2004; Mutschlecner et al., 2017; Rosemond et al., 2015) (Figure 1). These priming and nutrient effects were initially observed in terrestrial environments during the last century (Bingemann et al., 1953; Blagodatsky & Richter, 1998; Broadbent, 1947; Jenkinson et al., 1985; Löhnis, 1926), but until the last decade, they had been largely untested in aquatic environments (Bianchi, 2011; Guenet et al., 2010; Marín‐Spiotta et al., 2014). Recent aquatic priming studies have produced conflicting results, detecting aquatic priming effects in some environments (Bianchi et al., 2015; Guenet et al., 2014) but not others (Bengtsson et al., 2015; Catalán et al., 2015; Textor et al., 2018, 2019). The prevalence of priming and nutrient effects in high‐latitude aquatic ecosystems remains an important source of uncertainty in estimates of the magnitude and timing of the permafrost climate feedback (Abbott, Jones, et al., 2016; Holmes et al., 2008; Keuper et al., 2020; Tank et al., 2020; Textor et al., 2019; Wickland et al., 2012).

While priming and nutrient effects have been considered independently, even in the few studies where both were measured (Guenet et al., 2014; Hotchkiss et al., 2014; Jenkinson et al., 1985), they are likely functionally linked, at least in oligotrophic and mesotrophic ecosystems (Figure 1). In the absence of external nutrient inputs from humans or other sources (Brahney et al., 2014; Frei et al., 2020), DOM mineralization is the proximate source of nutrients in terrestrial and aquatic ecosystems (Fork et al., 2020; McDowell et al., 2006; Mutschlecner et al., 2017), meaning that increased DOM biodegradability may increase nutrient availability. In turn, nutrient‐rich environments support higher rates of primary productivity, root exudation, and production of more decomposable organic matter (Carey et al., 2019; Guenet et al., 2010; Hewitt et al., 2018; Mutschlecner et al., 2017; Salmon et al., 2018), increasing BDOC availability (Figure 1). The linkage between priming and nutrient effects complicates the interpretation of observational and experimental studies that only quantify one of the effects, because responses attributed to nutrients could be due to priming, and vice versa (Chen et al., 2019; Danger et al., 2013; Rosemond et al., 2015). Additionally, strong seasonal variation in nutrient concentrations and DOM properties can complicate comparison across studies (Holmes et al., 2008; Kortelainen et al., 2020; Wickland et al., 2012).

**Figure 2:**
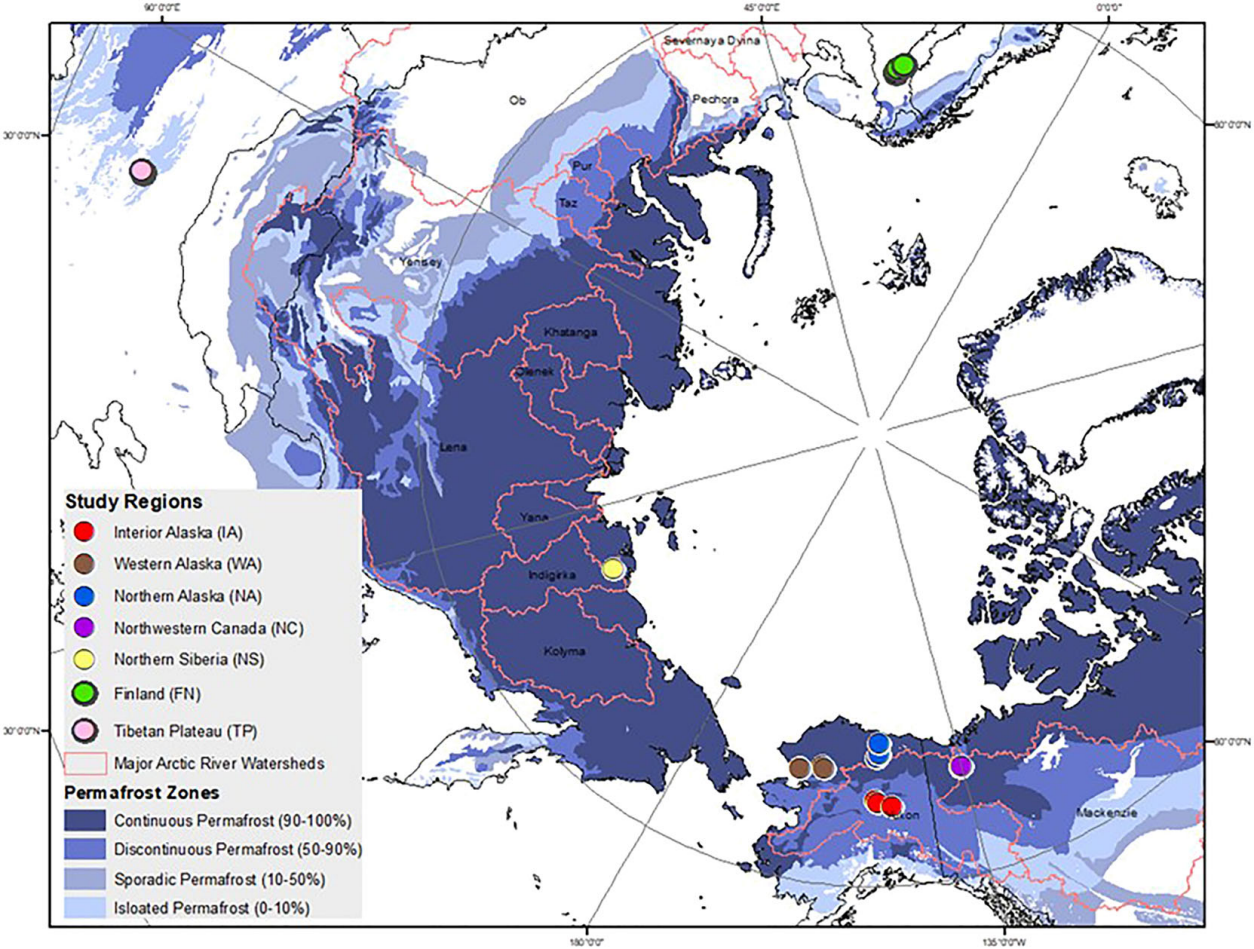
Map of sampling locations (colored circles), major Arctic watersheds, and permafrost distribution (Brown et al., 2002). See Table 1 for site details.

Here, we report results from an international experiment where we investigated the prevalence of priming and nutrient effects in 23 permafrost‐zone streams from seven high‐latitude or high‐altitude regions. We sampled streams in the late summer in Alaska, Canada, Siberia, Finland, and the Tibetan Plateau (Figure 2 and Table 1). Our primary objectives were to (1) identify large‐scale patterns of DOM molecular composition and metabolic stability in stream ecosystems across the permafrost zone, (2) quantify how additions of BDOC and nutrients affect modern DOM mineralization in diverse permafrost‐zone ecosystems, and (3) test how stream network position affects DOM dynamics. Based on findings from terrestrial and aquatic priming studies in other regions (Chen et al., 2019; Dorado‐García et al., 2015; Guenet et al., 2014), we hypothesized that the effects of BDOC and nutrient addition (i.e., the magnitude of priming and nutrient effects) would depend on background DOM composition and availability of nutrients. Following observations and hypotheses about DOM dynamics in high‐latitude stream networks (Cory et al., 2014; Drake et al., 2015; Vonk, Tank, Mann, et al., 2015; Zarnetske et al., 2018), we hypothesized that DOC biolability would decrease longitudinally (i.e., larger rivers would have lower BDOC) because of longer transport times in soils and in‐stream photodegradation and biodegradation (Catalán et al., 2016; Connolly et al., 2018; Creed et al., 2015; Shogren et al., 2019). We predicted larger priming and nutrient effects at sites with more stable DOC and lower nutrient availability, respectively. To test these predictions, we measured DOC disappearance in 28‐day incubations with and without acetate and inorganic nutrient additions. We quantified priming and nutrient effects by measuring acetate and background DOC drawdown and by characterizing background DOM composition by fluorescence spectroscopy and Fourier transform ion cyclotron resonance mass spectrometry (FT‐ICR MS).

**Table 1:** Site Characteristics

Region	Site code^a^	Latitude (DD)	Longitude (DD)	Permafrost zone	Dominant vegetation type	Geologic substrate and texture	Watershed contributing area (km^2^)	MAT (°C)/MAP (mm)	Major River Network
Interior Alaska	IA1	65.8007	−149.4392	Discontinuous	Black Spruce, moss, lichen, and low shrub	Loess (fine)	14	−7.5/270 (Ewing et al., 2015; Koch et al., 2014)	Yukon
	IA2	65.6540	−149.0921				12		
	IA3	65.3440	−146.9110			Loess, colluvium (mixed)	4		
Western Alaska	WA1	67.4830	−162.2150	Continuous	Sedge, dwarf shrub, and moss tundra	Glacial alluvium (coarse)	37	−5/300–400 (O'Donnell, Aiken, Butler, et al., 2016)	Noatak
	WA2	67.4740	−162.2250				748		
	WA3	67.7510	−158.1150	Continuous	Sedge, low shrub, and moss wetland	Glaciolacustrine (stratified‐mixed)	1,165		
	WA4	67.8450	−158.3160				2,860		
Northern Alaska	NA1	68.6867	−149.0975	Continuous	Tussock‐sedge, dwarf shrub, and moss tundra	Glacial till, loess (fine)	76	−10/320 (Abbott et al., 2015)	Sagavanirktok
	NA2	68.8772	−148.8445				3,604		
	NA3	69.6299	−148.6514				9,369		
Northwestern Canada	NC1	67.2517	−135.2716	Continuous	Black Spruce	Glacial till (fine)	5	−7.3/146 (Littlefair et al., 2017)	Peel
	NC2	67.3133	−135.1683				40		
	NC3	67.3360	−134.8714				71,658		
Northeastern Siberian	NS1	70.8317	147.5173	Continuous	Tussock‐sedge, dwarf and low shrub, and moss tundra	Silt (fine)	<1	−10.5/212 (Iwahana et al., 2014)	Indigirka
	NS2	70.8300	147.5118				122		
	NS3	70.8226	147.5135				14,600		
Finland	FN1	66.1478	26.1618	Non‐permafrost	Boreal forest/peatland/agriculture	Glacial till and peatlands (fine)	62	1.7‐2.8/560‐644 (de Wit et al., 2020; Lepistö et al., 2008; Mattsson et al., 2005)	Simojoki
	FN2	65.9529	25.9342				1,919		
	FN3	65.6618	25.0754				3,093		
Tibetan Plateau	TP1	37.4776	100.2885	Discontinuous	Swamp Meadow	Silt‐loam (fine)	4	−3.3/460 (Liu et al., 2018)	Shaliu
	TP2	37.4182	100.2392				32		
	TP3	37.3468	100.2259				420		

^a^ The sites in each region were nested within the same watershed, except Interior Alaska.

## 2 Methods

We collected stream samples from August to September 2016 from six regions across the permafrost zone and in September 2017 from one northern, non‐permafrost region (Figure 2). Water samples were collected from three or more locations within each study region. Regions were selected to include Arctic, boreal, and alpine ecosystem types and to represent a range of current and future climatic conditions in the permafrost zone (continuous, discontinuous, and non‐permafrost; Table 1). Samples were collected during late summer when background DOC biolability is typically lowest (Holmes et al., 2008; Mu et al., 2017; Wickland et al., 2012) and when permafrost DOC and nutrients are most likely to influence aquatic ecosystems during the period of maximum annual thaw (Abbott et al., 2014, 2015; Treat et al., 2016). To test our hypothesis about longitudinal (upstream‐downstream) patterns in DOM composition (Cory et al., 2014; Drake et al., 2015; Shogren et al., 2019), we selected sites that were nested in river networks, except in interior Alaska, where the three sites came from independent streams because of accessibility considerations. Our naming convention was a two‐letter acronym of the region name and a number starting from the smallest catchment (Table 1). In western Alaska, where we had two sets of nested catchments, WA1 was upstream of WA2 in one network, and WA3 was upstream of WA4 in a second network.

### 2.1 Characterization of Study Regions

For each region, we characterized a suite of ecological characteristics. We first delineated contributing watershed areas in ArcGIS (most sites) or Google Earth (Tibetan Plateau sites). For each watershed, we determined physical and textural properties of lithologic substrate using maps of surficial geology and descriptions from the literature (Hamilton, 2003; Kokelj et al., 2017; Yang et al., 2010). We classified each watershed as continuous permafrost (>90% of land surface underlain by permafrost), discontinuous permafrost (50–90%), or permafrost free (Brown et al., 1997). Lastly, we identified dominant vegetation types with the Circumpolar Arctic Vegetation Map (Walker et al., 2018) or referenced literature (Table 1).

The seven sampled regions include broad variation in climate, geology, topography, and vegetation (Table 1). Mean annual temperature (MAT) ranges from −5°C to −10.5°C across the permafrost‐affected sites and is 1.7°C to 2.8°C at the non‐permafrost sites in Finland. Mean annual precipitation (MAP) ranges from 140 to 600 mm (Table 1). In all permafrost‐affected regions, various types of permafrost degradation have been observed, including thermokarst and thermo‐erosional features such as retrogressive thaw slumps, thermo‐erosional gullies, and thermokarst lakes (Aanderud et al., 2019; Farquharson et al., 2019; Littlefair & Tank, 2018; Liu et al., 2019; Luo et al., 2019; Mu et al., 2019; Olefeldt et al., 2016). Other forms of less visible permafrost warming and degradation are also occurring in the study areas, including active‐layer thickening and talik (pockets of permanently thawed material) formation (Biskaborn et al., 2019; Shiklomanov et al., 2010; Vonk, Tank, Bowden, et al., 2015). Permafrost degradation across all the regions is projected to accelerate in the coming decades (Guo et al., 2012; Jafarov et al., 2018; Nicolsky et al., 2017; Romanovsky et al., 2010; Turetsky et al., 2020; Wang et al., 2020).

Though permafrost degradation is present in all the studied permafrost catchments, three of the seven regions (Canada, interior Alaska, and the Tibetan Plateau) were specifically chosen for their proximity to abrupt thaw features. The northwestern Canada Sites NC1 and NC2, which are underlain by glacial tills, drain watershed areas downstream of a large thaw slump—Site “FM3” in other studies (e.g., Littlefair et al., 2017). Site NC3 is located on the mainstem of the larger Peel River, which receives inputs from numerous slump‐affected tributaries (Kokelj et al., 2017; Littlefair & Tank, 2018; Zolkos et al., 2018). The interior Alaska Sites IA1 and IA2, which occur in thick, ice‐rich Pleistocene silt (Yedoma), are adjacent to a thawing pingo and thermokarst channel, respectively (Ewing et al., 2015; Koch et al., 2013). Site IA3 is a gravel bedded stream draining a partially burned watershed with limited loess cover and some isolated thermokarst features (Koch et al., 2014). The Tibetan Plateau sites are downstream of a thermo‐erosional gully in a silt‐dominated alpine swamp meadow (Chen et al., 2018; Liu et al., 2018). Even for these thaw‐adjacent sites, the current contribution of permafrost DOM and nutrients in the sampled streams is likely small because of dilution (Abbott et al., 2015; Larouche et al., 2015). However, we included sites in areas of actively degrading permafrost because thermokarst‐prone areas contain approximately half of permafrost‐zone organic matter and may contribute more than half of the permafrost climate feedback (Olefeldt et al., 2016; Turetsky et al., 2020).

The Siberian Site NS1 is a small pond that is connected to the river network via surface flow during high flows, while Sites NS2 and NS3 are part of the mainstem downstream of NS1 (Dean et al., 2020). The watersheds in western Alaska are underlain by continuous permafrost but differ with respect to topography and permafrost soil properties. Sites WA1 and WA2 drain an alpine watershed underlain by ice‐poor permafrost and parent material composed of glacially derived gravel and cobble substrate (O'Donnell, Aiken, Butler, et al., 2016). WA3 and WA4 drain an ice‐rich watershed with numerous thermokarst lakes and slumps in the catchment, though not adjacent to the sampling sites (O'Donnell, Aiken, Butler, et al., 2016). The northern Alaska sites occur in Arctic tundra and are underlain by continuous permafrost. NA1 is a stream called Oksrukuyik Creek that drains moist tundra with numerous thermokarst lakes in the catchment (Shogren et al., 2019). NA2 and NA3 are on the mainstem Sagavanirktok River, which is fed mostly by glacial runoff from the Brooks Range (Abbott et al., 2014; Cory et al., 2014; Hamilton, 2003). The Finnish sites represent a non‐permafrost region in the subarctic characterized by boreal forest and peatlands (Lepistö et al., 2008; Mattsson et al., 2005), providing an analog for future conditions in much of the permafrost zone.

### 2.2 Sample Collection and Incubation Setup

For the incubation, we followed the standardized protocol proposed by Vonk, Tank, Mann, et al. (2015), with minor modifications to suit the field conditions and laboratory analyses (SI: detailed protocol). Incubations were performed locally by each regional team, and samples were shipped to centralized locations for analysis (details in section 2.3). Stream water was filtered on site (0.7 μm, Whatman GF/F), and refrigerated until laboratory incubations were initiated. Incubations were started within 1 or 2 days after sample collection except for the western Alaska (WA) and the northeastern Siberian (NS) sites, which were initiated one week after sample collection due to field constraints.

For incubations, we divided the filtered bulk stream samples into 200‐ml aliquots and treated each aliquot with one of eight acetate (CH_3_COO^−^) and nutrient treatments (Table S1; Abbott & Ewing, 2020). We used acetate as the priming substrate in these experiments for three reasons: (1) Acetate is a highly biolabile form of DOC often used in ecosystem manipulations (Robbins et al., 2017; Zarnetske et al., 2011); (2) it is easily measurable via ion chromatography, allowing us to directly quantify disappearance of added substrate and background DOC (Baker et al., 1999; Hotchkiss et al., 2014); and (3) acetate naturally accumulates in permafrost during anaerobic metabolism and is released during permafrost thaw, representing up to a quarter of total permafrost DOC in some areas (Drake et al., 2015; Ewing et al., 2015; Neumann et al., 2016). We used ammonium (NH_4_^+^), nitrate (NO_3_^−^), and phosphate (PO_4_^3−^) as the inorganic nutrient substrates. These inorganic nutrients are commonly used in nutrient enrichment studies (Rosemond et al., 2015; Slavik et al., 2004), and they are released during permafrost degradation (Abbott et al., 2015; Keuper et al., 2017). We set treatment levels of acetate and nutrients (Table S1) based on observed concentrations of low‐molecular weight DOC and inorganic nutrients in streams draining thermokarst features (Abbott et al., 2014, 2015; Drake et al., 2015; Ewing et al., 2015; Tanski et al., 2017). The three acetate‐only treatment levels (A1, A2, and A3) had 1, 5, and 10 mg C L^−1^ of added acetate, respectively. The three nutrient‐only treatment levels (N1, N2, and N3) had a 25:5:1 blend of different concentrations of NH_4_^+^, NO_3_^−^, and PO_4_^3−^ (Table S1), based on observed ratios of these nutrients in thermokarst outflows (Abbott et al., 2015). The remaining two treatments were a high acetate plus high nutrient treatment (AN) and a control sample (CT) in which only deionized water was added. Though background DOC and nutrient conditions varied among the sites, we kept the treatments consistent for comparison and to simulate how permafrost degradation may release concentrations of acetate and nutrients uncorrelated with modern in‐stream conditions (Coch et al., 2020; Ewing et al., 2015; Tanski et al., 2017). While we recognize that micronutrients can limit microbial activity, we limited the experiment to carbon, nitrogen, and phosphorus additions for logistical reasons—for example, there is great diversity of observed micronutrients in permafrost waterways and soils (Carey et al., 2019; Krickov et al., 2020; Reyes & Lougheed, 2015).

We incubated water from each site in 24 borosilicate 250‐ml brown glass bottles (three replicates of the eight treatments), which were generally kept stationary on a benchtop at each incubation location. Incubations were done in the dark at room temperature (20°C), constraining DOC loss to biotic rather than photic processes. The relatively coarse filtration (0.7‐μm effective pore size) prior to incubation allowed ambient aquatic microorganisms to pass through the filter into the incubation bottles and mineralize the DOC (Dean et al., 2018; Larouche et al., 2015; Vonk, Tank, Mann, et al., 2015). Treatments were added only at the start of the incubations to simulate mixing of permafrost thaw products with modern DOM in stream networks (Abbott et al., 2015; Drake et al., 2015; Shogren et al., 2019; Tanski et al., 2017).

The incubation samples from the Tibetan Plateau sites were destroyed during shipping, but the background chemistry, optical samples, and molecular samples survived.

### 2.3 Background Chemistry, DOC, and Acetate Analyses

Incubation and baseline chemistry samples were collected, frozen, and sent for analysis at the Environmental Analytical Laboratory in the Department of Land Resources and Environmental Sciences at Montana State University (MSU). Inorganic nutrients (NH_4_^+^, NO_3_^−^, NO_2_^−^, and PO_4_^3−^) in unamended (background) stream waters were determined at μg L^−1^ levels on a QuAAtro39 continuous segmented flow analyzer (Seal Analytical, Inc.). We calculated dissolved inorganic nitrogen (DIN) as the sum of NH_4_^+^, NO_3_^−^, and NO_2_^−^. Acetate and other dissolved solutes in the treated incubation samples (NO_3_^−^, NO_2_^−^, and Cl^−^) were measured at mg L^−1^ levels on an ICS 2100 Ion Chromatograph (Dionex, Thermo Scientific) equipped with an anion column (ASX‐18 column). DOC and total nitrogen (TN) in all samples were determined using a V‐TOC CSH Total Carbon Auto‐Analyzer with a TNM‐1 Total Nitrogen Module (Shimadzu Corporation). Analytical uncertainties were determined for each instrument based on replication of samples and results for several representative working standards (supporting information). Samples were kept frozen at MSU until analysis, and values were only accepted if uncertainty was less than 10%. When concentrations were below the limit of detection, we set their values to half the detection limit.

### 2.4 Optical Properties and Molecular Composition of DOM

We collected additional subsamples at t_0_ and t_28_ from a subset of the treatments (CT, A3, and AN) for optical analysis via fluorescence spectroscopy. These subsamples were filter sterilized (0.22 μm, PES) into 40‐ml amber glass vials and stored in the dark at 4°C during shipment and until analysis at Utah State University within four months of arrival (Baker & Lamont‐BIack, 2001). We measured the absorbance and fluorescence of these subsamples with a spectrofluorometer (Aqualog, Horiba Scientific, Edison, New Jersey). We analyzed the absorbance data and the excitation emission matrices (EEMs) to calculate several common indices of DOM composition (Fellman, Spencer, et al., 2010; Kellerman et al., 2018; McKnight et al., 2001; Weishaar et al., 2003), including colored DOM (CDOM; absorbance at 254 nm), biological index (BIX), humification index (HIX), fluorescence index (FI), peak T (tryptophan‐like) to peak C (fulvic/humic‐like) index (TC), and specific ultraviolet (UV) absorbance at 254 nm (SUVA_254_) (Gabor, Baker, et al., 2014; Gabor et al., 2015). Because the behavior of the ambient DOC was our primary focus, we subtracted the measured acetate concentration from total DOC concentration for the acetate‐addition treatments before calculating SUVA_254_ and other metrics (e.g., priming as described in section 2.5). All samples were corrected for inner filter effects, Rayleigh scatter, and blank subtraction in MATLAB™ (version 6.9; MathWorks, Natick, Massachusetts), and samples that exceeded 0.3 absorbance units at excitation 254 nm were diluted with deionized water to be under 0.3 absorbance units and re‐run (reported values have been adjusted proportionally to the dilution factor).

We selected a subset of samples for analysis of DOM chemical composition via ultrahigh resolution mass spectrometry with a 21 T FT‐ICR MS (Hendrickson et al., 2015; Smith et al., 2018). Because of the high cost of these analyses, we selected only the CT and A3 treatments at the t_0_ and t_28_ time steps for a subset of sites (a total of 33 samples). These subsamples were filtered to 0.7 μm (GF/F pre‐combusted at 450°C for 5 hr) to remove potential flocculation and stored frozen in pre‐leached, high‐density polyurethane bottles until analysis at the National High Magnetic Field Laboratory, Tallahassee, FL (Spencer et al., 2015; Textor et al., 2019). We used DOC concentration to calculate the appropriate volume for solid phase extraction (100‐mg Bond Elut PPL, Agilent Technologies) following the method described by Dittmar et al. (2008) and aimed for a concentration of 40 μg C ml^−1^ for DOM extracts eluted with 1 ml of methanol. All FT‐ICR MS samples were analyzed in negative ion mode and molecular formulae were examined in the mass range of 170–1,500 m/z and reassigned in PetroOrg Software (Corilo et al., 2013, 2016; Liu et al., 2016). We examined elemental combinations of C_1‐45_ H_1‐92_N_0‐4_O_1‐25_S_0‐2_ with mass errors less than 300 ppb and excluding noise signals >6*σ* root‐mean‐square (RMS) baseline (O'Donnell, Aiken, Butler, et al., 2016). Elemental stoichiometries and modified aromaticity indices (AI_mod_) (Koch & Dittmar, 2016) were used to assign molecular formulae into seven different compound classes using a script developed by Hemingway (2018): *unsaturated phenolic low O/C* = AI_mod_ < 0.5, H/C < 1.5, O/C < 0.5; *unsaturated phenolic high O/C* = AI_mod_ < 0.5, H/C < 1.5, O/C ≥ 0.5; *polyphenolic* = AI_mod_ 0.50–0.67; *condensed aromatic* = AI_mod_ > 0.67; *aliphatic* = H/C ≥ 1.5, O/C < 0.9; *N* = 0; *sugar‐like* = O/C > 0.9; and *peptide‐like* = H/C ≥ 1.5, O/C < 0.9 *N* ≥ 1 (O'Donnell, Aiken, Swanson, et al., 2016). Although molecular peaks detected during FT‐ICR‐MS may represent multiple isomers, we interpret DOM composition based on the relative abundance of molecular formulae assigned to each compound class. Therefore, molecular formulae assigned to the same compound class may herein be collectively described as compounds.

### 2.5 Biodegradability, Priming, and Nutrient Effects

Hereafter, we refer to “background” DOC as the total DOC concentration minus added and ambient acetate. To calculate rates of acetate and background DOC consumption, we poured off and froze ~15 ml of sample into polypropylene vials immediately following the addition of treatments (t_0_), after 7 days (t_7_), and after 28 days (t_28_). We calculated change in background DOC and acetate for each replicate individually as the proportional difference between the t_0_ and t_7_ or t_0_ and t_28_ concentrations (e.g., a ΔDOC_7_ of −0.2 represents a 20% decrease in DOC concentration or a BDOC value of 20% after 7 days). We then calculated the mean and standard deviation of ΔDOC and ΔAcetate across the three replicates for each site and time step. Replicates with evidence of contamination or analytical error were excluded from the means. We calculated change in optical properties (ΔOptical) and relative abundance (ΔRA) of molecular composition in the same way as ΔDOC and ΔAcetate.

We calculated priming and nutrient effects for each site as the ΔDOC in each treatment minus the ΔDOC in the unamended (control) treatment. This yielded positive values for the nutrient and priming effects when the treatment resulted in greater background DOC consumption (i.e., positive priming) and negative values when the treatment DOC consumption was less than the control (i.e., negative priming), following the typical sign convention (Bianchi et al., 2015; Guenet et al., 2010; Hotchkiss et al., 2014).

### 2.6 Statistical Analyses

To test for differences in ΔDOC, ΔAcetate, ΔOptical, and ΔRA among treatments, we used one‐way analysis of variance (ANOVA) with site as a blocking factor to account for non‐independence (Malone et al., 2018). We tested if priming effects were different across time steps for the various treatments with a two‐way ANOVA, also with site as a blocking factor. For all ANOVAs, we used Tukey's honest significant difference post hoc tests for multiple comparisons with a Bonferroni correction and a decision criterion of *α* = 0.05. We evaluated normality, homoscedasticity, and leverage (Cook's distance) for each test visually. To evaluate if nutrient and priming effects were significant (i.e., statistically different than 0), we used two‐sided *t* tests for each treatment, interpreted after a Bonferroni correction.

To evaluate similarity of DOM compounds and optical properties across sites, we used principal component analysis (PCA). We scaled all parameters to have a mean of 0 and a variance of 1, and we transformed the non‐normally distributed parameters to avoid undue influence from extreme observations and to allow the computation of parametric probability ellipses (Kotz & Nadarajah, 2004). We used the Kaiser criterion, scree plots, and the percent explained variance to decide how many principal components to report (Horikoshi et al., 2020; Wickham et al., 2020).

To test our hypotheses about links between priming and nutrient effects, we used Spearman rank correlations (*ρ*) to quantify relationships (including non‐linear relationships) among BDOC, priming/nutrient effects, and background nutrient chemistry. We evaluated relationships with background DOC, DIN, PO_4_^3−^, and the molar ratios of those parameters (DOC:DIN, DOC:PO_4_^3−^, and DIN:PO_4_^3−^), using a decision criterion of *α* = 0.05. Finally, we used Spearman rank correlations to test for links between water chemistry, climate variables (i.e., MAT and MAP), and watershed area.

All statistical analyses were performed in R.3.5.0 (R Core Team, 2018).

## 3 Results

### 3.1 Ambient Stream Chemistry and DOM Properties

**Figure 3:**
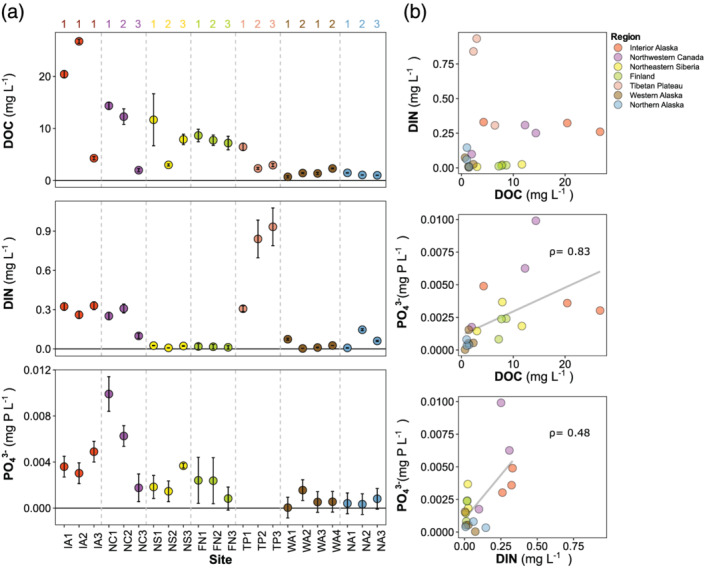
Background nutrient concentrations by site and region. (a) The mean (± standard deviation of three replicates) of dissolved organic carbon (DOC), dissolved inorganic nitrogen (DIN), and phosphate (PO_4_^3−^) in streams from the seven study regions. Regions are separated by the vertical dashed lines and are ordered from highest to lowest mean DOC (left to right). Within regions, sites are ordered by increasing watershed size (longitudinal stream position indicated by the numbering system above the panels) within nested networks, except for IA, where the sites were independent, and WA, where there are two networks. (b) Biplots of mean background nutrient concentrations with Spearman correlation coefficients (*ρ*) shown when significant (*p* < 0.05). Phosphate was not determined for TP sites because of sample loss during shipping.

The ambient concentrations of DOC, DIN, and PO_4_^3−^ at the time of sampling varied widely across the sites and regions (Figure 3). DOC ranged from 0.7 to 27 mg L^−1^ and was higher in smaller streams relative to larger rivers for five of the six regions with longitudinally nested sampling locations (Figure 3a). DOC was negatively correlated with watershed area across sites (*ρ* = −0.51, *n* = 22). The highest DOC concentration occurred at the thermokarst‐affected sites in interior Alaska and northwestern Canada (14–27 mg L^−1^). DIN was uncorrelated with DOC (Figure 3b; *ρ* = 0.38, *n* = 22) and showed a variety of downstream patterns. NO_3_^−^ constituted 65% of DIN on average, but the relative abundance of DIN species (i.e., NO_3_^−^, NO_2_^−^, and NH_4_^+^) varied across sites (supporting information: Background), with NO_3_^−^ ranging from 6.6% at NC1 to 99% of DIN at WA1. NH_4_^+^ and NO_2_^−^ constituted 26% and 8.8% of DIN on average. PO_4_^3−^ was strongly correlated with DOC and to a lesser extent DIN (*ρ* = 0.83 and 0.48, respectively, *n* = 19), with several sites at or near the detection limit (Figure 3). Nutrient concentrations were not correlated with MAT across sites, but DIN and PO_4_^3−^ were negatively correlated with MAP (*ρ* = −0.49 and −0.52, *n* = 22 and 19, respectively).

**Figure 4:**
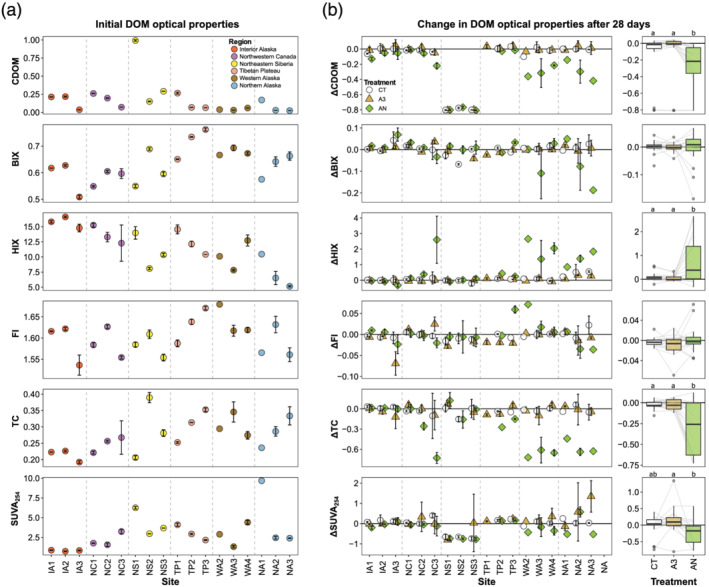
Initial optical properties of dissolved organic matter (DOM) and change through time during the incubations. (a) Initial colored DOM (CDOM), biological index (BIX), humification index (HIX), fluorescence index (FI), peak T to peak C index (TC), and specific UV absorbance at 254 nm (SUVA_254_). (b) Proportional change during the incubation for the three treatments where optical proxies were measured: control (CT), high acetate (A3), and acetate plus nutrients (AN). colored dissolved organic matter (CDOM), biological (mean *n* = 3). for the three treatments where optical measurements were made. In panels (a) and (b), symbology follows Figure 3. Boxplots show the quartiles, median, minimum, and maximum within 1.5 times the interquartile range (IQR) and outliers beyond 1.5 times the IQR. The faint lines behind the boxplots link sites to aid interpretation.

Optical properties of DOM showed as much variability within regions as among them (Figure 4a). There were two initial SUVA_254_ values that exceeded 5 L mg^−1^ m^−1^ (Figure 4a), potentially indicating iron, pH, or NO_3_^−^ interference (Weishaar et al., 2003). There were general decreases in CDOM and HIX moving downstream within regions (Figure 4a), and both parameters were correlated negatively with watershed area across sites (*ρ* = −0.46 and −0.71, respectively, *n* = 18). BIX and TC increased moving downstream for most regions and were positively correlated with watershed area across sites (*ρ* = 0.47 and 0.72, respectively, *n* = 18). FI and SUVA_254_ showed mixed patterns across sites (Figure 4a). CDOM and BIX were correlated with MAP (*ρ* = −0.62 and 0.58, respectively, *n* = 18), and SUVA_254_ was negatively correlated with MAT (*ρ* = −0.48, *n* = 18).

There were numerous positive and negative correlations among DOM optical properties and molecular composition (Figure S1), with particularly strong relationships (|*ρ*| > 0.65) between polyphenolics and CDOM (*ρ* = 0.79, *n* = 30), polyphenolics and BIX (*ρ* = −0.75, *n* = 30), unsaturated phenolics (low O/C) and CDOM (*ρ* = −0.68, *n* = 30), and aliphatics and HIX (*ρ* = −0.66, *n* = 30). Molecular composition and to a lesser extent optical properties were also correlated with background nutrient concentrations (Figure S1). DOC and PO_4_^3−^ had particularly strong relationships with many parameters, though DIN was also correlated with most molecular composition parameters (Figure S1).

**Figure 5:**
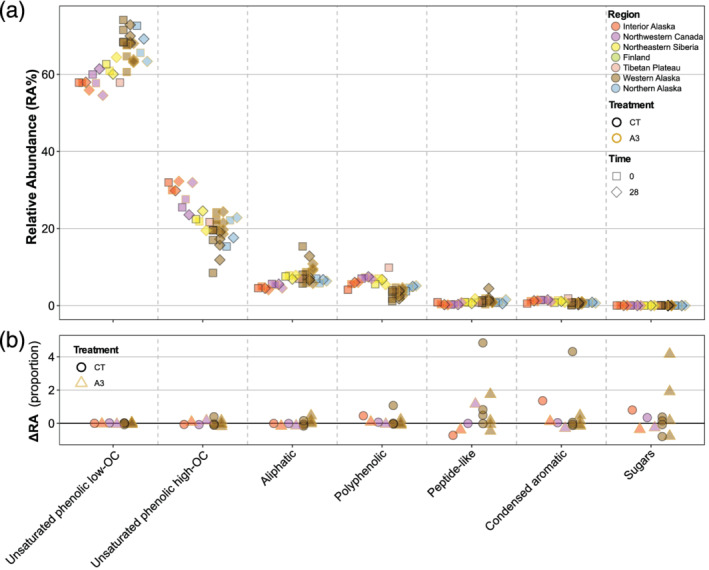
Molecular composition of DOM and change in relative abundance of different components. (a) The relative abundance of composition classes Initial and Final (t_0_ and t_28_). (b) The change in relative abundance expressed as a proportion.

DOM molecular composition was surprisingly similar across regions and sites (Figure 5), in contrast with the high inter‐ and intra‐regional variability in concentrations and bulk optical properties described previously. Despite differences in climate, watershed area, and vegetation, there was strong compositional consistency across sites (Figure 5a). The most abundant compound class was unsaturated phenolics (low O/C), which ranged from 58% to 75% across sites. Unsaturated phenolics (high O/C) were the second most abundant compound class for all but one site in western Alaska (WA1), ranging from 8.5% to 32%. Aliphatics made up less than 9% of the relative abundance for all but one site (also WA1). Polyphenolics and other compounds made up less than 10% of the relative abundance across sites (Figure 5a). DOM molecular compounds were not generally correlated with climate variables, except that polyphenolics were negatively correlated with MAT and MAP (*ρ* = −0.70 and −0.88, *n* = 11), and unsaturated phenolics were correlated with MAP (*ρ* = 0.67 and −0.67 for low and high O/C, respectively, *n* = 11).

**Figure 6:**
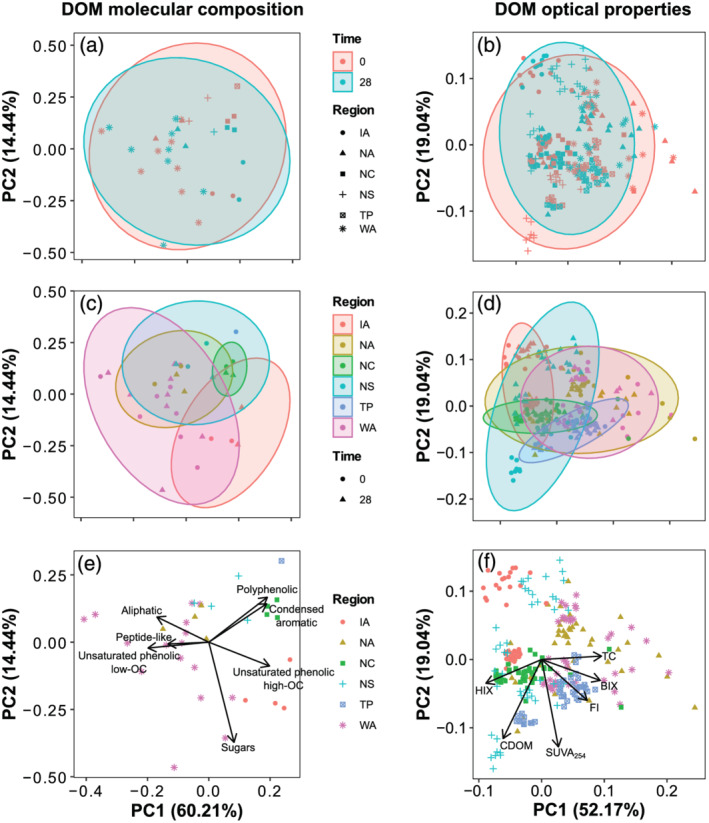
Principal component analysis of the molecular composition and optical properties of DOM. The percentage of variance explained by the first and second principal components (PC1 and PC2) is shown in parentheses next to the axis titles. Parametric probability ellipses are drawn for the indicated groups (i.e., time of sampling in panels a and b and regional provenance of the sample in panels c and d). Loadings for each parameter are shown in panels (e) and (f). PC3 explained 11% of the variation (Figure S2).

For the multivariate analysis of molecular composition and optical properties, the first three principal components of the PCA explained 86% of the variation in molecular composition and 87% of the variation in optical properties across regions, treatments, and time steps (Figures 6 and S2). The probability ellipses overlapped for all regions, supporting the univariate results of compositional and optical similarity among regions (i.e., greater intra‐region than inter‐region variability; Figures 6c and 6d).

### 3.2 DOC Biodegradability and Effects of Priming and Nutrients

**Figure 7:**
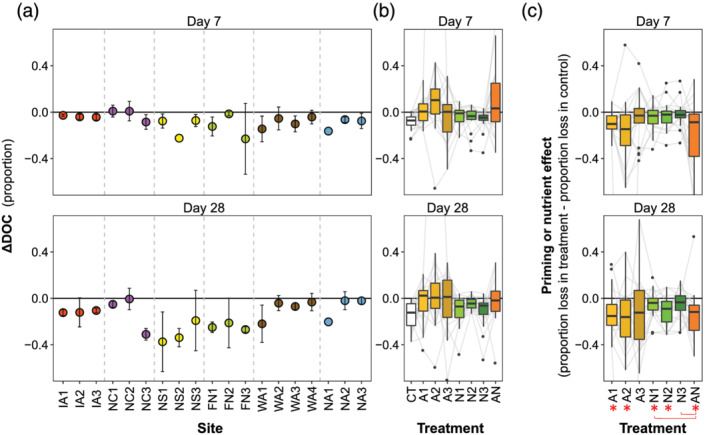
Change in DOC concentration (ΔDOC) after 7 and 28 days and effects of nutrient and acetate addition. (a) Proportional ΔDOC in unamended control treatments by site. (b) Distribution of ΔDOC by treatment. (c) The priming or nutrient effect by treatment expressed as the difference between ΔDOC in the amended treatment and ΔDOC in the unamended control. A positive value represents more DOC loss in the amended treatment (i.e., positive priming or nutrient effect), while a negative value indicates less DOC loss. Asterisks below the *x* axis indicate statistical difference from 0 based on two‐sided *t* tests (Bonferroni‐adjusted *p* < 0.05), and brackets show statistically significant contrasts among treatments based on Tukey‐HSD comparisons following ANOVA (Bonferroni‐adjusted *p* < 0.05). Symbology follows Figure 4.

The 7‐ and 28‐day incubations revealed relatively low DOC biodegradability but substantial variation among sites (Figures 7, S3, and S4). In the unamended control treatment, proportional change in DOC ranged from −0.01 to −0.22 after 7 days (median and mean ΔDOC_7_ = −0.06 and −0.08) and −0.03 to −0.52 after 28 days (median and mean ΔDOC_28_ = −0.9 and −0.16; Figure 7a). ΔDOC_7_ and ΔDOC_28_ were positively correlated (*ρ* = 0.54, *n* = 19). The downstream pattern in ΔDOC for sites in nested stream networks differed by region (Figure 7a), and neither ΔDOC_7_ nor ΔDOC_28_ was significantly correlated with watershed area (*ρ* = −0.27 and 0.10, respectively, *n* = 19), MAT (*ρ* = 0.05 and 0.01, *n* = 19), or MAP (*ρ* = −0.30 and −0.03, *n* = 19). In the control treatment, ΔDOC_7_ was correlated with numerous DOM optical properties and molecular compounds, particularly aliphatics, unsaturated phenolics (high O/C), and HIX (Figure S1). However, ΔDOC_28_ was only correlated with FI (Figure S1). Similarly, control ΔDOC_7_ was correlated with background DOC, DIN, and PO_4_^3−^ (*ρ* = 0.61, 0.69, and 0.65, respectively), but ΔDOC_28_ was not (Figure S1).

The effects of added acetate and nutrients (i.e., priming and nutrient effects) were not statistically different at 7 and 28 days (*F*_1,239_ = 0.15, *p* = 0.69), so we pooled estimates from both time steps before testing for significance (Figure 7c). We observed negative priming and nutrient effects (i.e., less decrease in DOC) in five of the seven treatments compared to the unamended control (Figure 7c; Bonferroni‐corrected *p* values for each treatment's *t* test: A1 = 0.0005, A2 = 0.03, A3 = 0.15, N1 = 0.03, N2 = 0.004, N3 = 0.19, and AN = 0.001 0.03, 0.004, and 0.001). Priming and nutrient effects differed by treatment (*F*_1,239_ = 3.97, *p* = 0.0009), with the treatments including acetate showing consistently stronger suppression, reducing median mineralization of background DOM to at or near zero (Figures 7b and 7c). Priming and nutrient effects also differed by site (*F*_1,239_ = 4.63, *p* = 1.0 × 10^−8^), with variation in both magnitude and sign of these effects for individual sites (Figures S4 and S5).

**Figure 8:**
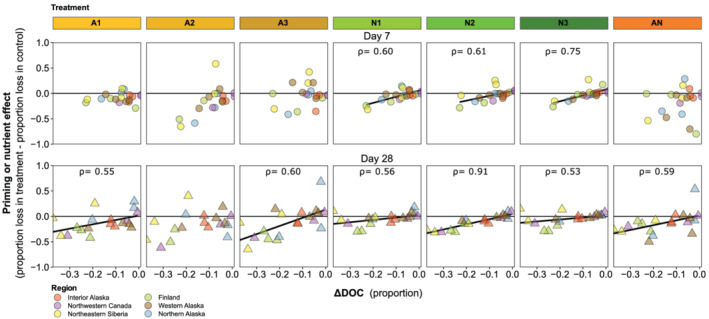
Relationships between ΔDOC in the unamended control treatment and priming or nutrient effects. Positive priming or nutrient effects indicate more DOC loss in the treatment than in the control (i.e., positive priming). Spearman correlation coefficients (*ρ*) shown when significant (*p* < 0.05). The linear fit lines are shown for convenience, though the Spearman analysis does not assume linearity.

The effect of nutrient addition was consistently correlated with ΔDOC across time steps (Figure 8), with neutral or positive nutrient effects at sites with low DOC biodegradability (i.e., ΔDOC ≈ 0) but negative nutrient effects at sites with high DOC biodegradability. The effect of acetate addition was only correlated with ΔDOC for two of the six treatment by time step combinations (Figure 8). The combined effect of nutrient and acetate addition (i.e., the AN treatment) was not correlated with ΔDOC_7_ but was correlated with ΔDOC_28_, with consistently negative effects at sites with biodegradable DOC but neutral to positive effects at sites with lower DOC biodegradability (Figure 8).

### 3.3 Changes in Acetate, DOM Optical Properties, and DOM Composition

**Figure 9:**
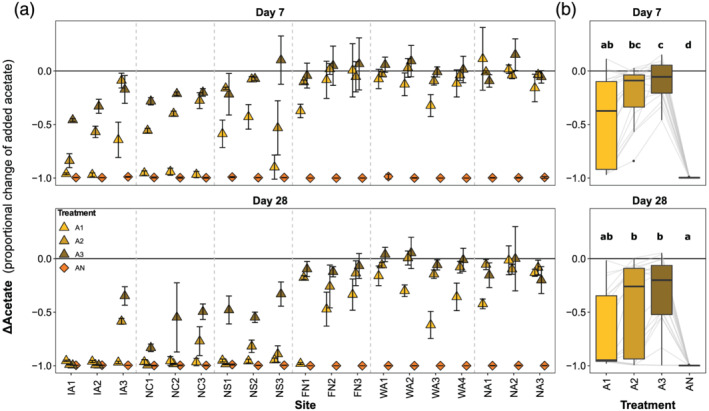
Change in added acetate after 7 and 28 days. (a) Proportional change in added acetate (ΔAcetate) by site. (b) Distribution of ΔAcetate by treatment. Differences in the letters show statistically significant contrasts among treatments based on Tukey‐HSD comparisons following ANOVA (Bonferroni‐adjusted *p* < 0.05). Symbology follows Figure 4.

**Figure 10:**
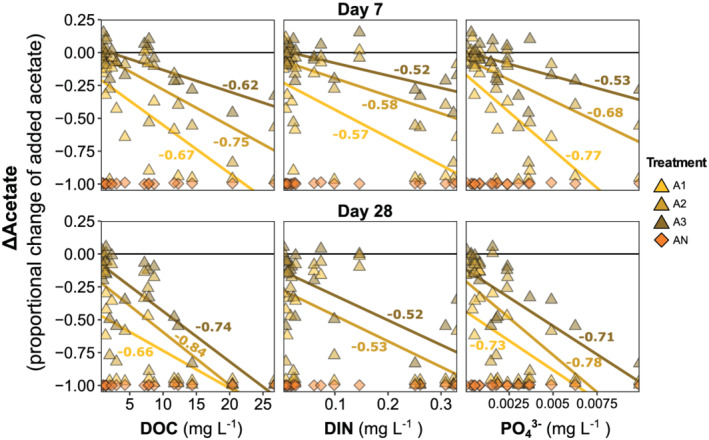
Relationships between change in added acetate and background nutrients. Spearman correlation coefficients (*ρ*) for each treatment shown when significant (*p* < 0.05). Symbology follows Figure 8.

Though we expected complete consumption of added acetate in all treatments, actual ΔAcetate varied in sign and magnitude (Figures 9 and S6). Both the incubation and background data suggested that acetate uptake was nutrient limited. First, acetate uptake was complete in the AN treatment (ΔAcetate range of −0.98 to −1.0), where nutrients were added with acetate (Figure 9). Second, acetate uptake was lower for higher acetate treatments (e.g., the |ΔAcetate| for A3 < A1), suggesting increasing stoichiometric constraints (Figure 9a). Third, there were strong and consistent correlations between background nutrients and ΔAcetate for A1, A2, and A3, with more acetate uptake in sites with higher background nutrient concentrations (Figure 10). ΔAcetate was more strongly correlated with background DOC and PO_4_^3−^ than with DIN (Figure 10). There were no significant correlations between ΔAcetate and nutrient ratios (DOC:DIN, DOC:P‐PO_4_^3−^, and DIN:P‐PO_4_^3−^) in the acetate amended treatments (Figure S7). For the treatments without acetate addition (i.e., CT, N1, N2, and N3), ΔAcetate was generally negative but highly variable (Figure S6), largely because initial unamended acetate concentration was extremely low across sites (mean ± SD = 0.07 ± 0.1 mg C L^−1^).

DOM optical properties (which were measured at t_0_ and t_28_ for treatments CT, A3, and AN) showed both general and site‐specific trends over the course of the experiment (Figures 4 and S8). Across sites, CDOM and TC decreased through time for all three treatments, with significantly larger decreases in the AN treatment (Figure 4b). ΔTC did not show consistent relationships with background chemistry, but ΔCDOM was associated with DIN, with more CDOM loss at sites with lower DIN, lower DIN:P‐PO_4_^3−^, and higher DOC:DIN (Figure S9). SUVA_254_ decreased and HIX increased during the experiment, but only for the AN treatment (Figure 4b). ΔHIX was consistently negatively correlated with background DOC and PO_4_^3−^, with greater increases in HIX at lower concentrations (Figure S9). Like for CDOM, ΔSUVA_254_ in the AN treatment was consistently correlated with DIN, with greater decreases in SUVA_254_ at sites with lower t_0_ DIN, higher DOC:DIN, and lower DIN:P‐PO_4_^3−^ (Figure S9). Across optical properties, the lower DOC sites (i.e., western and northern Alaska) tended to show greater responses to the AN addition (Figure 4b). The northeastern Siberia sites showed distinct CDOM and SUVA_254_ dynamics, with large decreases in both properties regardless of treatment (Figure 4b).

DOM molecular composition (which was measured at t_0_ and t_28_ for a subset of the CT and A3 treatments) was remarkably stable across the incubations (Figure 5b). The median change in relative abundance was less than 0.05 across all compounds, though there were larger increases or decreases in some of the low‐abundance classes, particularly peptide‐like compounds and sugars (Figure 5b). However, these changes were largely associated with low initial concentrations and were not consistently associated with treatment (Figure 5b).

The multivariate analysis showed little systematic change in molecular composition and optical properties across treatments (Figures 6a/6b and S2a/S2b). However, individual sites did show substantial shifts, and optical properties showed a slight homogenization (a tighter or smaller probability ellipse) over the course of the experiment (Figures 6b and S2b), in line with the univariate results.

## 4 Discussion

In this study, we investigated how BDOC and nutrients from thawing permafrost could influence the stability of background DOM in waterways of the permafrost zone. Using optical and molecular characterization techniques in combination with a DOM biodegradability experiment, we found that DOM from across the permafrost zone had surprisingly similar molecular composition, dominated by unsaturated phenolics, but different optical properties and biodegradability. Contrary to our hypotheses, the addition of BDOC and nutrients suppressed the mineralization of background DOM, and there were no systematic differences in DOM biodegradability based on stream network position. These experiments comprise one of the most geographically diverse investigations of aquatic priming to date and our results have several implications for carbon and nutrient balance in the permafrost zone. We discuss the implications and limitations of these findings below.

### 4.1 DOM Is More Than the Sum of Its Compounds

The similarity in molecular composition of DOM from across permafrost regions supports a growing body of evidence that the building blocks of DOM are similar across freshwater and marine environments (Danczak et al., 2020; Kellerman et al., 2018; Zark & Dittmar, 2018). Because the sources of aquatic DOM vary strongly across ecosystems and through time (e.g., soil, vegetation, and microorganisms), this similarity has mainly been attributed to “filter effects” that select for certain compounds at the dissolution phase and during transport (Gabor, Eilers, et al., 2014; Marín‐Spiotta et al., 2014; Roth et al., 2019; Zark & Dittmar, 2018; Zarnetske et al., 2018). A non‐exclusive hypothesis for this convergence is that autochthonous production of DOM by aquatic autotrophic and heterotrophic organisms contributes new DOM with a common “aquatic” signature (Harjung et al., 2018; Kellerman et al., 2018; Lee‐Cullin et al., 2018). These filtering and aquatic contribution hypotheses have been supported by DOM optical analysis, which shows initial diversity associated with biogeochemical origin followed by convergence toward a more or less universal aquatic DOM signature (Coble et al., 2019; Gabor, Eilers, et al., 2014; Mutschlecner et al., 2018; Wünsch et al., 2019).

This compositional and optical homogeneity of aquatic DOM contrasts with its structural and functional diversity. A wide range of functional experiments and observations have revealed that DOM differs in its interaction with inorganic nutrients, light sensitivity, compound‐specific reactivity, and availability to microorganisms in different physico‐chemical conditions (Abbott, Jones, et al., 2016; Cory et al., 2014; Dean et al., 2020; Drake et al., 2015; Mu et al., 2017; Nalven et al., 2020; Vonk, Tank, Mann, et al., 2015; Wymore et al., 2015). Even in our relatively limited experiment with filtered, late‐summer DOM from cold regions, we observed biodegradability that ranged nearly twentyfold (i.e., 3% to 52%) and priming and nutrient responses that varied in size and sign.

**Figure 11:**
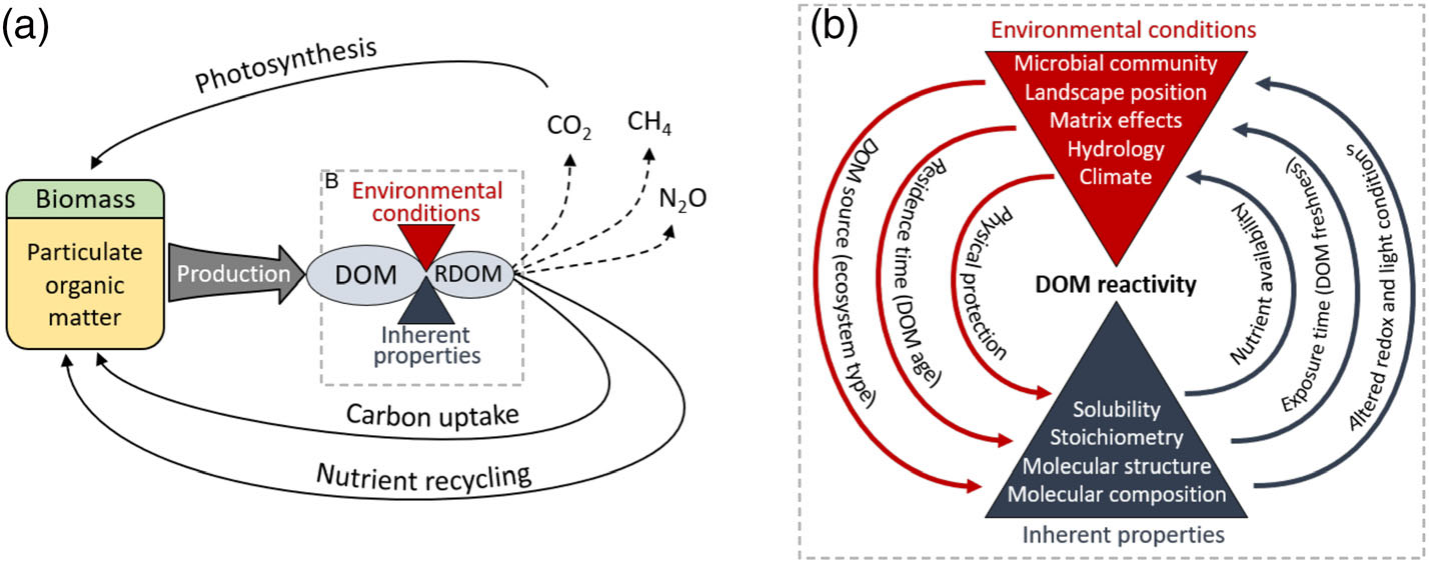
Conceptual model of the carbon cycle emphasizing the role of reactive dissolved organic matter (RDOM) as a choke point or regulator. (a) While biomass and particulate organic matter contain most of the Earth's organic carbon, the pool of reactive DOM as determined by environmental and inherent factors regulates biogeochemical processes such as respiration, nutrient mineralization, methanogenesis, and denitrification. (b) The amount of reactive DOM at a given moment in space and time depends on two‐way interactions among environmental conditions and DOM properties. Together, these dynamics regulate the persistence and processing (biotic and abiotic) of DOM. The curved arrows provide examples of links between DOM properties and environmental conditions. The inherent factors that influence DOM degradability are both cause and consequence of the environmental factors that interact to determine realized reactivity (actual rates of DOM alteration, assimilation, or mineralization).

The functional diversity of DOM despite apparent compositional homogeneity implies either that our measures of DOM composition are inadequate (Gabor, Baker, et al., 2014; Hawkes et al., 2020; Simon et al., 2018) or that additional factors regulate DOM persistence and broader ecological function (Figure 11). In either case, this disconnect is problematic because DOM composition is routinely used as a proxy for reactivity, toxicity, and bioavailability (Abbott, Baranov, et al., 2016; Kaiser et al., 2017; Zhang et al., 2019). Additionally, this disconnect suggests that the hope of finding easily measured yet generally applicable proxies of DOM stability—long a goal of aquatic ecosystem science (Balcarczyk et al., 2009; Fellman, Hood, & Spencer, 2010; McDowell et al., 2006)—may continue to be elusive. Both optical and molecular approaches are routinely criticized for ambiguous interpretations, high cost or time investment, and comparability issues among equipment (Benk et al., 2018; Gabor, Baker, et al., 2014; Hawkes et al., 2020; Kellerman et al., 2018; Ruhala & Zarnetske, 2017; Simon et al., 2018). Similarly, empirical measures of DOM function, such as the experiment presented here, have their own suite of limitations. Filtration and isolation of samples from their biotic and abiotic context can demonstrate what is biogeochemically possible rather than what is ecologically relevant (Hanson et al., 2011; James & Boone, 2005; Kothawala et al., 2012). Ultimately DOM characterization, whether optical or molecular, will be most enlightening when coupled with independent measures of DOM function, such as incubations (Cory et al., 2013; Helton et al., 2015; Vonk, Tank, Mann, et al., 2015), in situ processing studies (Ewing et al., 2015; Fork et al., 2020; Hall et al., 2016; Harjung et al., 2018; Judd et al., 2006; Mineau et al., 2016), isotope probing experiments (Kellerman et al., 2018; Mulholland, 2004), and characterization of microbial dynamics (Danczak et al., 2020; Guenet et al., 2010; Nalven et al., 2020).

### 4.2 Intrinsic and Extrinsic Factors Regulate DOM Reactivity Across the Terrestrial‐Aquatic Continuum

The perennial debate about intrinsic and extrinsic controls on organic matter stability has extended over decades and across environments from soil to sea (Arnosti, 2004; Ewing et al., 2006; Kellerman et al., 2015; Marín‐Spiotta et al., 2014; Schmidt et al., 2011). Until recently, the dominant paradigm was that inherent properties of organic matter determined decay rate, with secondary effects from environmental factors such as temperature, redox, and microbial community (Allison, 2006; Kalbitz et al., 2003; Kleber et al., 2011; Weintraub & Schimel, 2003). Recent observations have challenged this “DOM quality” paradigm, with biodegradable organic matter persisting for centuries in some environments while recalcitrant organic matter is broken down on sub‐yearly timescales in others (Arnosti, 2004; Ewing et al., 2006; Kalbitz et al., 2005; Marín‐Spiotta et al., 2014; Schmidt et al., 2011). Furthermore, decomposer communities appear to be biochemically omnipotent, breaking down any organic compounds given the right conditions and enough time (Arnosti, 2011; Jaffé et al., 2013; Manzoni et al., 2012; Sinsabaugh et al., 2015). Based on these observations, a new paradigm has been proposed that considers organic matter persistence as an ecosystem property, emphasizing environmental controls such as pH, redox, temperature, light, and physical protection by the soil or water matrix as the determinants of organic matter persistence (Kaiser & Kalbitz, 2012; Lehmann & Kleber, 2015; Marín‐Spiotta et al., 2014; Schmidt et al., 2011). According to this ecosystem property hypothesis, the reactivity of organic matter is a function of external factors and has little to do with the initial nutrient content or inherent molecular structure of the organic matter itself.

Both the inherent and ecosystem hypotheses of DOM stability overlook an important internal feedback: DOM concentration and properties are major determinants of many of the environmental conditions that modulate DOM stability in actual ecosystems (Figures 1 and 11). For example, DOM abundance and reactivity influence pH, redox, microbial community, light penetration, nutrient supply, and priming effects (Battin et al., 2008; Fork et al., 2020; Kellerman et al., 2015; Manzoni et al., 2012; Pinay et al., 2015; Zarnetske et al., 2012). These feedbacks (Figure 11) create spatially and temporally dynamic relationships between DOM composition and expressed reactivity (Catalán et al., 2016; Helton et al., 2015; Wymore et al., 2015). Consequently, it is the interaction between inherent DOM composition and ecosystem conditions that determines the relative importance of reaction rates and exposure times for a particular compound (Abbott, Baranov, et al., 2016; Frei et al., 2020; Kolbe et al., 2019; Oldham et al., 2013). For example, in our dark incubations, the effect of added nutrients or BDOC depended on the decomposability of the background DOM (Figure 8), potentially due to kinetic and nutrient constraints (Guenet et al., 2010; Wymore et al., 2015). However, these responses could vary under different light and nutrient conditions in actual ecosystems, where DOM can act as a nutrient source and light attenuator (Fanta et al., 2010; Fork et al., 2020; Nalven et al., 2020; Rodríguez‐Cardona et al., 2016).

These concepts of interactive stability and context‐dependent reactivity (Abbott, Baranov, et al., 2016; Kolbe et al., 2019) mean that causal relationships between DOM structure and stability cannot be quantified by correlating DOM characteristics with ecosystem properties such as residence time or climate, though such conclusions are routinely inferred in molecular, optical, and ecosystem studies (Catalán et al., 2016; Cory et al., 2014; Kellerman et al., 2015). This approach does not answer the questions: Did the DOM persist because of its composition and structure, or does it have that structure and composition because it persisted? Similarly, experiments that quantify inherent reactivity isolated from environmental interactions with nutrients and new DOM have limited power to establish causality. We again emphasize that experiments quantifying DOM structure, reactivity, and sensitivity to priming and nutrient effects are needed to establish the relative importance of inherent and ecosystem controls on DOM reactivity in different environments (Danczak et al., 2020; Fork et al., 2020; Guenet et al., 2014; Hotchkiss et al., 2014; Mutschlecner et al., 2017; Nalven et al., 2020; Textor et al., 2019).

### 4.3 Consequences for Permafrost‐Zone Carbon and Nutrient Budgets

The overall negative priming and nutrient effects we observed suggest that permafrost‐derived DOC and nutrients are not likely to destabilize modern DOM in high‐latitude rivers, lakes, and estuaries. Most high‐latitude DOM flux occurs during the snowmelt period, while most permafrost‐derived nutrient and DOM release happens in the late thaw season (Abbott et al., 2014; Holmes et al., 2008; Raymond et al., 2007; Spencer et al., 2009; Treat et al., 2016). This means that priming and nutrient effects are unlikely to alter annual net ecosystem carbon balance in permafrost waterways. However, priming and nutrient substrate from degrading permafrost could have important seasonal impacts, depending on background DOM biolability (Figure 8). The few permafrost‐zone studies that have quantified seasonal DOM biolability have generally found higher biolability during snowmelt, though some locations experience little seasonal variation, and others have higher biolability in the winter (Abbott et al., 2014; Holmes et al., 2008; Larouche et al., 2015; Mann et al., 2012; Mu et al., 2017; Mutschlecner et al., 2018; Wickland et al., 2012). Permafrost‐derived nutrients and BDOC could substantially alter aquatic food webs in the fall and winter by suppressing biolabile DOM processing and stimulating consumption of less biolabile DOM (Figure 8).

While the effects of permafrost‐derived material may have seasonally constrained impacts on bulk background DOM, CDOM and other optically defined DOM pools responded strongly to nutrient and BDOC addition (Figure 4). CDOM is a fundamental control on light availability for primary production and penetration of damaging light in high‐latitude aquatic and marine ecosystems (Evincent et al., 1998; Fork et al., 2020; Matsuoka et al., 2015; Spencer et al., 2009; Stedmon et al., 2011). If the observed acceleration of CDOM breakdown by nutrients (Figure 4b) is general, the effects could be substantial on aquatic and marine productivity, riverine DOM flux, and microbial communities (Bonilla et al., 2009; Mann et al., 2016; Squires et al., 2002). While long‐term nutrient data are sparse for the permafrost zone, there are several indicators that nutrient availability may be increasing in aquatic ecosystems (Abbott et al., 2015; Frey & McClelland, 2009; Kendrick et al., 2018; Shogren et al., 2019). Dynamics including expansion of wildfire, shifts in vegetation, extension of the thaw season, thermokarst formation, and hydrological changes are all increasing the supply and potentially the delivery of nutrients to rivers and lakes in the permafrost zone (Abbott, Jones, et al., 2016; Carey et al., 2019; Hewitt et al., 2018; Rodríguez‐Cardona et al., 2020; Salmon et al., 2018; Tank et al., 2020; Treat et al., 2016). We note the need for further research on these CDOM effects because we did not measure change in optical properties in the nutrient only treatments (section 2). This means we cannot isolate whether nutrients alone or the combination of nutrients and BDOC accelerated CDOM breakdown, though the BDOC‐only treatment showed no change (Figure 4b).

Finally, the diversity of longitudinal patterns of DOM properties that we observed challenges the simplified view of reactive DOM in the headwaters and stable DOM in larger rivers (Cory et al., 2014; Drake et al., 2015; Mann et al., 2015; Prokushkin et al., 2011; Vonk, Tank, Mann, et al., 2015). While such a longitudinal decrease in DOM reactivity certainly exists in some watersheds during some parts of the year, broader spatial sampling suggests that headwaters have more diversity in DOM sources and properties compared to larger rivers rather than systematically more reactive DOM (Abbott et al., 2018; Shogren et al., 2019; Zarnetske et al., 2018). More generally, residence time in many permafrost river networks is on the order of days to weeks (Tank et al., 2020), limiting the time biotic and abiotic reactions can modify DOM, even compared with laboratory rates of DOM processing at elevated temperatures (Cory et al., 2013; Vonk, Tank, Mann, et al., 2015). Rather than invoking in‐stream processes, changes in DOM sources associated with vegetation, soil and sediment types, and catchment residence times (i.e., water travel time before reaching the channel) can create a variety of longitudinal patterns depending on local ecological context (Abbott et al., 2015; Kling et al., 2000; Tank et al., 2020), including increases, decreases, and convergence toward the watershed mean (Connolly et al., 2018; Neilson et al., 2018; Shogren et al., 2019).

## Supporting information



Supporting Information S1

Data Set S1

## Data Availability

Data and supplementary tables and figures are attached as supplemental information and are available as part of the Hydroshare archive for this work (Abbott & Ewing, 2020), which includes analytical code used to derive figures.
