# Supplementary material for: Stream Dissolved Organic Matter in Permafrost Regions Shows Surprising Compositional Similarities but Negative Priming and Nutrient Effects
Source: Global Biogeochem Cycles. 2021 Jan 11;35(1):e2020GB006719. doi: 10.1029/2020GB006719 (PMC7816262; doi:10.1029/2020GB006719)
Supplement: Supplementary file 1 — Supporting Information S1 [file GBC-35-e2020GB006719-s001.pdf]

Incubation and analysis of late season stream water from 23 locations within seven northern or high-elevation regions in Asia, Europe, and North America in late summer of 2016 and 2017

Supplementary information for:
Wologo, E., Shakil, S., Zolkos, S., Textor, S., and 23 co-authors. 2020. Stream dissolved organic matter in permafrost regions shows surprising compositional similarities but negative priming and nutrient effects. *Global Biogeochemical Cycles,* doi: 10.1029/2020GB006719.

Citation for Hydroshare resource that includes analytical source code:
Abbott, B. W. and S. A. Ewing. 2020. Incubation and analysis of late season stream water from 23 locations within seven northern or high-elevation regions in Asia, Europe, and North America in late summer of 2016 and 2017. HydroShare.
https://doi.org/10.4211/hs.cb522bbb0f6449ac9d6ca2c189e5c604.

Contents

Supplementary dataset 1
Additional methods 3
  S1.1. Site Descriptions 3
  S1.2. Experimental Treatments and Additional Analyses 4
  S1.3. Shimadzu V-TOC CSH 5
  S1.4. Dionex ICS-2100 Anion Column 6
  S1.5. Bibliography for Additional Methods........ 6
Supplementary tables........ 7
  Table S1: target concentrations of incubation treatments........ 7
Supplementary figures 8
  Figure S1: Spearman correlations among optical properties and molecular composition of DOM. 8
  Figure S2: Time series of acetate and DOC concentrations during incubations. 9
  Figure S3: Example time series of acetate and DOC concentrations during incubations. 10
  Figure S4: Proportional change in DOC concentration by site and treatment. 11
  Figure S5: Priming and nutrient effects by site........ 12
  Figure S6: Change in acetate by site and treatment........ 13
  Figure S7: Correlations between change in added acetate and background stoichiometry. 14
  Figure S8: DOM optical properties at t_0 and t_{28}. 15
  Figure S9: Relationships between change in optical properties and background chemistry. 16
Detailed protocol used by each regional team........ 17

Supplementary dataset

The file titled “Priming_Database.xlsx” contains six tabs:

1. *Sites*: Information including watershed size, climate characteristics, geology, and vegetation.
2. *Background*: Ambient (unamended) chemistry of each site at the time of sample collection.
3. *Incubations*: Dissolved organic carbon (DOC), acetate, and solute concentrations from incubations time series.
4. *Optical_Properties*: Optical proxies from spectrofluorometry.
5. *DOM_Composition*: Compounds and families of compounds from FT-ICR MS analysis.
6. *MetaData*: Brief description of units and data file.

Additional methods

S1.1. Site Descriptions

The northern Alaska sites are longitudinally connected on the Sagavanirktok River, north of the Brooks Range in Alaska (NA). A shallow active layer limits the vegetation in this zone to plants with shallow rooting depths such as small flowering plants, grasses and low growing shrubs. This region is within the continuous permafrost zone and has high soil organic carbon (SOC), greater than 35 kg / m^2 (Grosse et al., 2011; Tarnocai et al., 2009).

The interior Alaska sites include two ice-rich silt-dominated sites including a low order tributary of the Yukon River (IA1) and a low order tributary of Hess Creek (IA2), and a mixed lithology headwaters catchment in the Beaver Creek drainage (IA3). The Yukon and Hess Creek sites are dominated by late Pleistocene to Holocene loess deposits (fine grained sediments, wind transported, and deposited and colluvially reworked in unglaciated areas) (Muhs & Budahn, 2006; Schirrmeister et al., 2013) with high syngenetic ice content, termed “yedoma” (French & Shur, 2010; Kanevskiy et al., 2011; Schirrmeister et al., 2013).

The northwestern Canada (NC) sites consist of three longitudinally connected sites along a tributary to and main stem of the Peel River in the Northwest Territories, Canada (NC1-3), with underlying substrate of fine glacial till hosting permafrost with high ice content. Thermokarst features in this region are characterized by large thaw-slumps with long debris tongues that deliver sediments and nutrients to downstream waterways (Kokelj et al., 2013; Littlefair et al., 2017).

The western Alaska sites (WA) are divided into two sub-regions (WA1-2 and WA3-4). WA 1 and WA2 are in an alpine setting within the Noatak National Preserve, an area managed by the U.S. National Park Service. These sites are underlain by coarse, glacially derived cobbles prone to draining freely. The textural nature of these sites does not promote permafrost growth or preservation nor high levels of organic matter storage and accumulation giving these waters a “pristine” character and low DOC concentrations. These two stream sites are located within the Agashashok River, which flows into the

Noatak River. The other sub-region, WA3 and WA4 also lie within the Noatak National Preserve. WA3, the Imeleyak River flows downstream into the Cutler River (WA4) and ultimately into the Noatak. The WA3 and 4 sites are not comprised of the same coarse glacial alluvium as WA1 and 2. Rather these sites are underlain by permafrost and are more closely related to the northern Alaska sites (NA).

The northern Siberia (NS) sites are ice-rich and silt dominated (Dean et al., 2020; Weiss et al., 2016). Much of the syngenetic permafrost in Siberia was formed and deposited during the late Pleistocene in unglaciated regions of Siberia. However, these sites within the Indigirka River watershed drain younger Holocene materials and are dominated by thermokarst lakes and polygonal ice-wedge networks (Iwahana et al., 2014). These sites are in a low relief area and within proximity to one another (<1km). This small channel flows into the larger Indigirka River and eventually into the East Siberian Sea.

The Finland sites (FN) are located along the Simojoki River basin which traverses through a mosaic of peatlands (53%), conifer forests (40%), agricultural fields(3%), water (3%), and peat harvesting areas and urban developments (2%). These sampling locations represent a non-permafrost, peat environment and are much warmer receive more precipitation than the other sites. The uppermost sampling site is headwater system while the two other sites are located along the mainstem (24km from FN1 and 51km from FN2, respectively).

S1.2. Experimental Treatments and Additional Analyses

Stream water from each site was sampled in bulk (6 L), filtered (0.7 μm, Whatman GF/F precombusted 450°C > 5hrs) into acid-washed 1 L amber bottles, and refrigerated until laboratory incubations were initiated. Experimental treatments (Table S.1) consisted of varying levels of labile carbon (acetate), which has been shown to be a substantial portion of DOC in permafrost ice cores (Stephanie A Ewing et al., 2015), and inorganic nutrients (NH_4, NO_3, PO_4) known to be present in thaw waters (Abbott et al., 2015; Drake et al., 2015; S.A. Ewing et al., 2015; Wickland et al., 2018) and act as key electron donors and acceptors in soil-stream interfaces (Hedin et al., 1998). In addition to the solute subsamples, two 50 mL samples were collected in 60 mL Nalgene bottles at the initiation and

termination of the incubation experiment (0 and 28 days) for Fourier Transform Ion Cyclotron Resonance Mass Spectrometry (FT-ICR MS) analysis of the character and composition of the organic matter in solution. FT-ICR-MS samples were frozen immediately and stored at -40˚C until analysis.

S1.3. Shimadzu V-TOC CSH

Analytical uncertainty for DOC and TN measurements are based on the reproducibility of known standards and working standards throughout the analysis procedure. Analyses were organized by site in order to achieve the best possible calibration range for increased accuracy, precision, and sensitivity. Each study region was unique in the matrix effect (ambient solute concentrations) and could vary dramatically in terms of DOC and TN concentrations. Some regional groups that are not longitudinally connected expressed large differences in DOC concentrations between sites despite being regionally similar (IA1 DOC >> IA3 DOC), see Figure S.3.

Several standards were used to measure the uncertainty associated with each analysis. One consensus reference material (CRM) (Batch 11 Lot # 03-11) from the Hansell Laboratory at the University of Miami was used as reference for accuracy of our instruments relative to others who participated to the consensus. This CRM served as reference for both DOC and TN measurements, however, the concentrations present in this standard were much lower (0.5 mg C/L) than the DOC of several regions and often had an uncertainty up to 10% due to the high calibration range required for samples with acetate treatments. Standard_W, a well sample from Gallatin Valley, Montana was also used as a reference for both DOC and TN which is consistently 2.0 mg C/L and 3.0 mg N/L. A unique mixture of excess sample from the western Alaska sites was created to act as a representative standard for permafrost sites. This mixture was divided into two subsamples and one of which was spiked with acetate for a reference ion. Similar mixtures were prepared from excess northwestern Canada samples when the WA mixtures were depleted.

S1.4. Dionex ICS-2100 Anion Column

Select dissolved anion concentrations were measured using an ion chromatograph. Solutes quantified include acetate (CH_3COO^-), nitrate (NO_3^-), nitrite (NO_2^-), sulfate (SO_4^-) and chloride (Cl^-). Experimental design and analytical methodology prioritized obtaining accurate acetate measurements over other solutes in solution. Acetate readily adheres to the column before most other anions in solution, however the acetate peak clusters together with other un-identified organics at the beginning of the chromatograph. To separate these peaks and precisely quantify acetate, the flow of carrier KOH (potassium hydroxide) is set at slow rate to separate the clustered peaks. KOH concentration was kept low (12mM) until the acetate had eluted and then the KOH concentration was ramped (to 39mM) to speed elution of other analytes.

S1.5. Bibliography for Additional Methods

Abbott, B. W., Jones, J. B., Godsey, S. E., Larouche, J. R., & Bowden, W. B. (2015). Patterns and persistence of hydrologic carbon and nutrient export from collapsing upland permafrost. *Biogeosciences*, *12*(12), 3725–3740. https://doi.org/10.5194/bg-12-3725-2015

Dean, J. F., Meisel, O. H., Martyn Rosco, M., Marchesini, L. B., Garnett, M. H., Lenderink, H., et al. (2020). East Siberian Arctic inland waters emit mostly contemporary carbon. *Nature Communications*, *11*(1), 1627. https://doi.org/10.1038/s41467-020-15511-6

Drake, T. W., Wickland, K. P., Spencer, R. G. M., McKnight, D. M., & Striegl, R. G. (2015). Ancient low–molecular-weight organic acids in permafrost fuel rapid carbon dioxide production upon thaw. *Proceedings of the National Academy of Sciences*, *112*(45), 13946–13951. https://doi.org/10.1073/pnas.1511705112

Ewing, S.A., Paces, J. B., O'Donnell, J. A., Jorgenson, M. T., Kanevskiy, M. Z., Aiken, G. R., et al. (2015). Uranium isotopes and dissolved organic carbon in loess permafrost: Modeling the age of ancient ice. *Geochimica et Cosmochimica Acta*, *152*, 143–165. https://doi.org/10.1016/j.gca.2014.11.008

Ewing, Stephanie A, O'Donnell, J. A., Aiken, G. R., Butler, K., Butman, D., Windham-Myers, L., & Kanevskiy, M. Z. (2015). Long-term anoxia and release of ancient, labile carbon upon thaw of Pleistocene permafrost. *Geophysical Research Letters*, *42*(24), 10,730-10,738. https://doi.org/10.1002/2015GL066296

French, H., & Shur, Y. (2010). The principles of cryostratigraphy. *Earth Science Reviews*, *101*, 190–206. https://doi.org/10.1016/j.earscirev.2010.04.002

Grosse, G., Harden, J., Turetsky, M., Mcguire, A. D., Camill, P., Tarnocai, C., et al. (2011). Vulnerability of high - latitude soil organic carbon in North America to disturbance, *116*, 1–23. https://doi.org/10.1029/2010JG001507

Hedin, L. O., von Fischer, J. C., Ostrom, N. E., Kennedy, B. P., Brown, M. G., & Robertson, G. P. (1998). Thermodynamic constraints on Nitrogen transformations and other biogeochemical processes at soil-stream interfaces. *Ecology*, *79*(2), 684–703.

Iwahana, G., Takano, S., Petrov, R. E., Tei, S., Shingubara, R., Maximov, T. C., et al. (2014). Geocryological characteristics of the upper permafrost in a tundra-forest transition of the Indigirka River Valley, Russia. *Polar Science*, *8*(2), 96–113. https://doi.org/10.1016/j.polar.2014.01.005

Kanevskiy, M., Shur, Y., Fortier, D., Jorgenson, M. T., & Stephani, E. (2011). Cryostratigraphy of late Pleistocene syngenetic permafrost (yedoma) in northern Alaska, Itkillik River exposure. *Quaternary Research*, *75*(2011), 584–596. https://doi.org/10.1016/j.yqres.2010.12.003

Kokelj, S. V., Lacelle, D., Lantz, T. C., Tunnicliffe, J., Malone, L., Clark, I. D., & Chin, K. S. (2013). Thawing of massive ground ice in mega slumps drives increases in stream sediment and solute flux across a range of watershed scales. *Journal of Geophysical Research: Earth Surface*, *118*(2), 681–692. https://doi.org/10.1002/jgrf.20063

Littlefair, C. A., Tank, S. E., & Kokelj, S. V. (2017). Retrogressive thaw slumps temper dissolved organic carbon delivery to streams of the Peel Plateau, NWT, Canada. *Biogeosciences*, *14*, 5487–5505.

Muhs, D. R., & Budahn, J. R. (2006). Geochemical evidence for the origin of late Quaternary loess in central Alaska, *337*, 323–337. https://doi.org/10.1139/E05-115

Schirrmeister, L., Froese, D., Tumskoy, V., Grosse, G., & Wetterich, S. (2013). Yedoma: Late pleistocene ice-rich syngenetic permafrost of Beringia. In *Encyclopedia of Quaternary Science* (Vol. 3, pp. 542–552).

Tarnocai, C., Canadell, J. G., Schuur, E. A. G., Kuhry, P., Mazhitova, G., & Zimov, S. (2009). Soil organic carbon pools in the northern circumpolar permafrost region. *Global Biogeochemical Cycles*, *23*, 1–11. https://doi.org/10.1029/2008GB003327

Weiss, N., Blok, D., Elberling, B., Hugelius, G., Jørgensen, C. J., Siewert, M. B., & Kuhry, P. (2016). Thermokarst dynamics and soil organic matter characteristics controlling initial carbon release from permafrost soils in the Siberian Yedoma region. *Sedimentary Geology*, *340*, 38–48. https://doi.org/10.1016/j.sedgeo.2015.12.004

Wickland, K. P., Waldrop, M. P., Aiken, G. R., Koch, J. C., Jorgenson, M. T., & Striegl, R. G. (2018). Dissolved organic carbon and nitrogen release from boreal Holocene permafrost and seasonally frozen soils of Alaska. *Environmental Research Letters*, *13*(6), 065011. https://doi.org/10.1088/1748-9326/aac4ad

Supplementary tables

Table S1: target concentrations of incubation treatments

Treatment	Acetate (mg C/L)	Ammonium (mg N/L)	Nitrate (mg N/L)	Phosphate (mg P/L)
A1	1.0			
A2	5.0			
A3	10.0			
N1		0.5	0.1	0.02
N2		2.5	0.5	0.1
N3		5.0	1.0	0.2
AN	10.0	5.0	1.0	0.2
CT				

Note. Stock solutions were prepared and delivered to 200mL incubation samples using a 1mL auto-pipettor. Values in table represent the final concentration increase of each treatment.

Supplementary figures

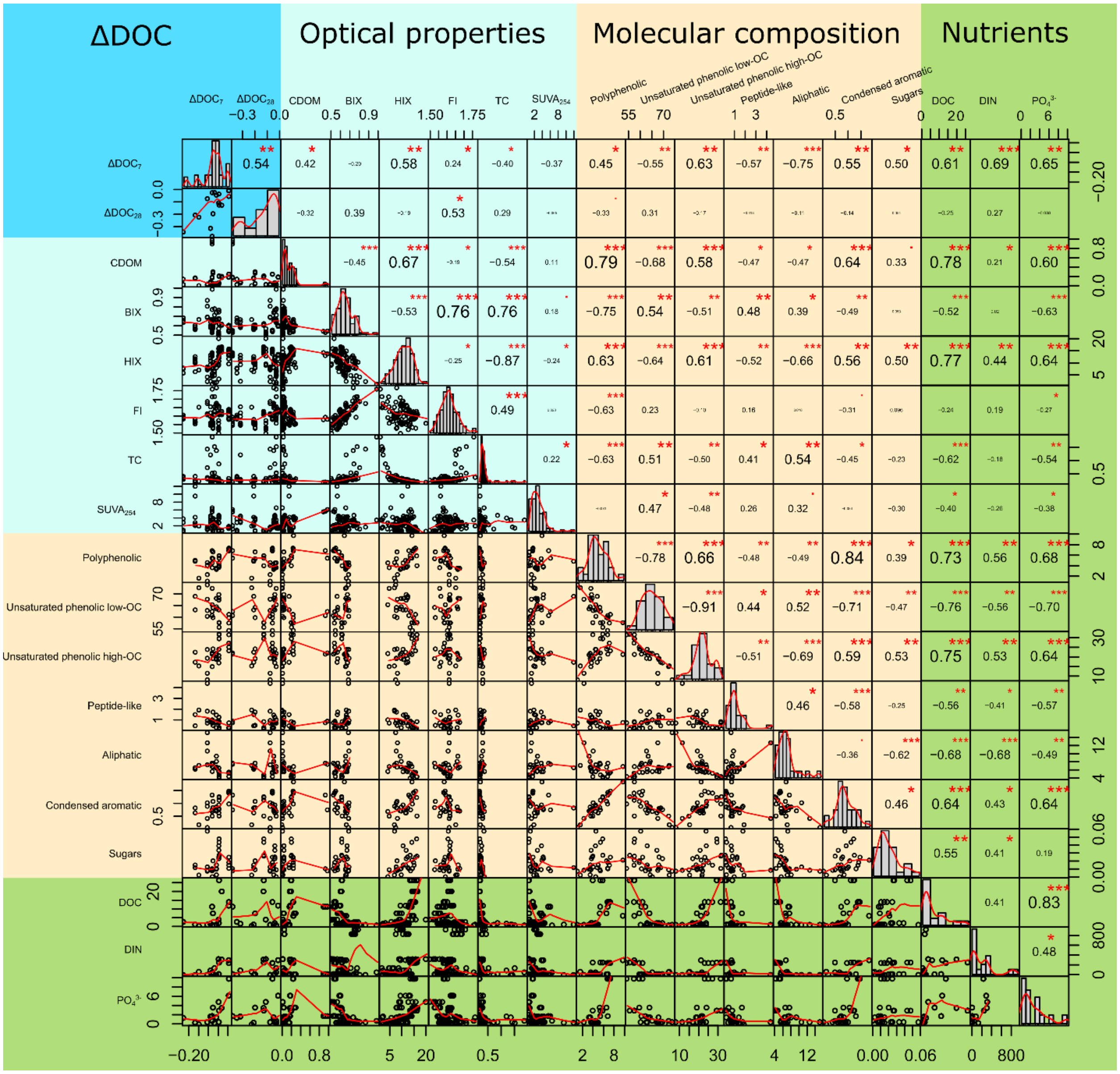

Figure S1: Spearman correlations among BDOC, optical properties, and molecular composition of DOM. Biodegradable DOC (ΔBDOC) is shown for the unamended control treatment for 7 and 28 days. The diagonal tiles show histograms for each parameter. Bivariate scatter plots are shown on the bottom left half of the matrix with a moving average fit line. Strength of the Spearman correlation is shown on the upper right half of the matrix, with red symbols indicating statistical significance at the α = 0.1 (.), 0.05 (*), 0.01 (**), and 0.001 (***) levels. For convenience, the blue and tan shading colors group the parameters into those associated with DOC biodegradability, optical properties, and molecular composition.

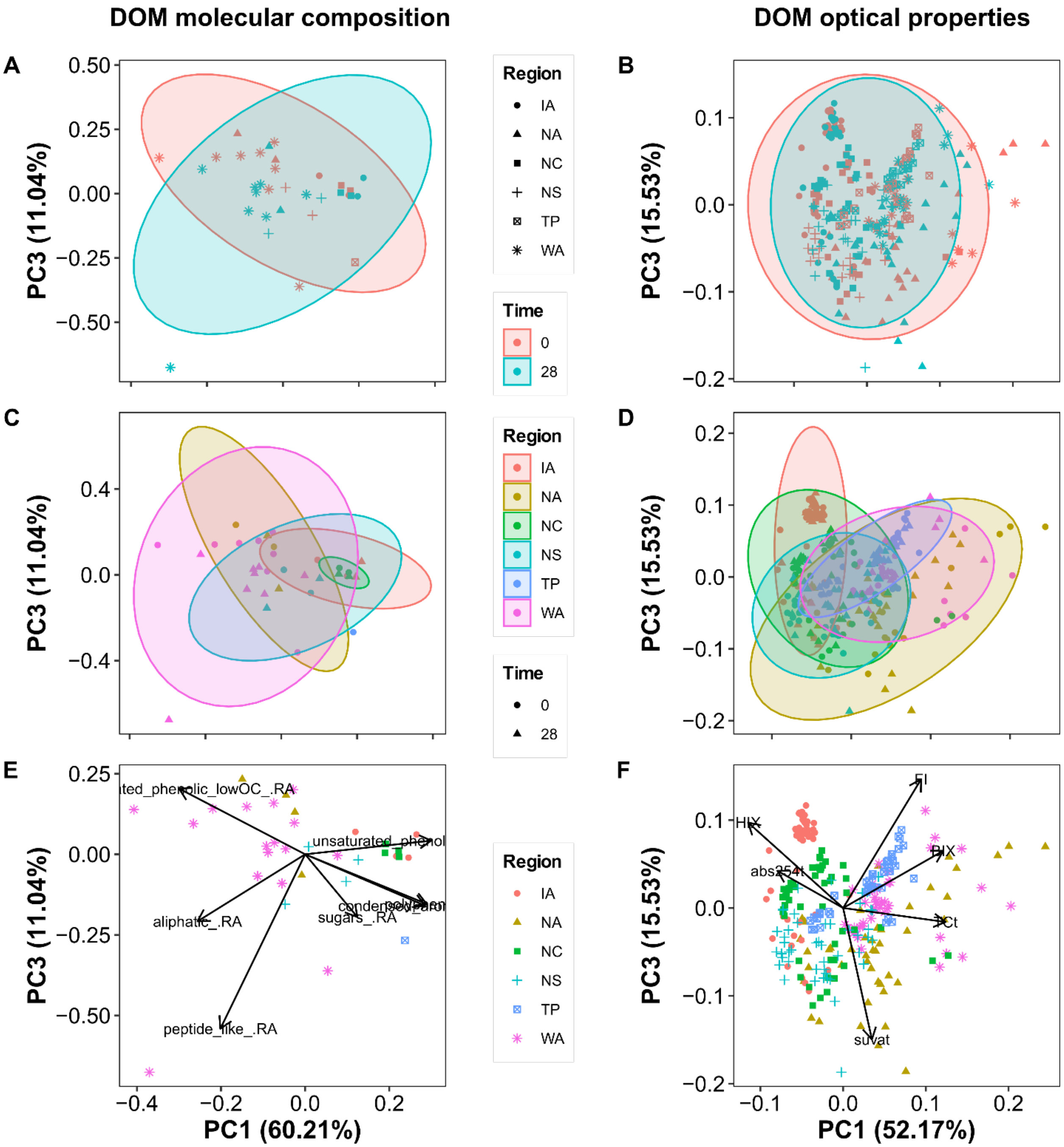

Figure S2: Principal component analysis of the molecular composition and optical properties of DOM. Symbology follows Figure 6 in the main manuscript.

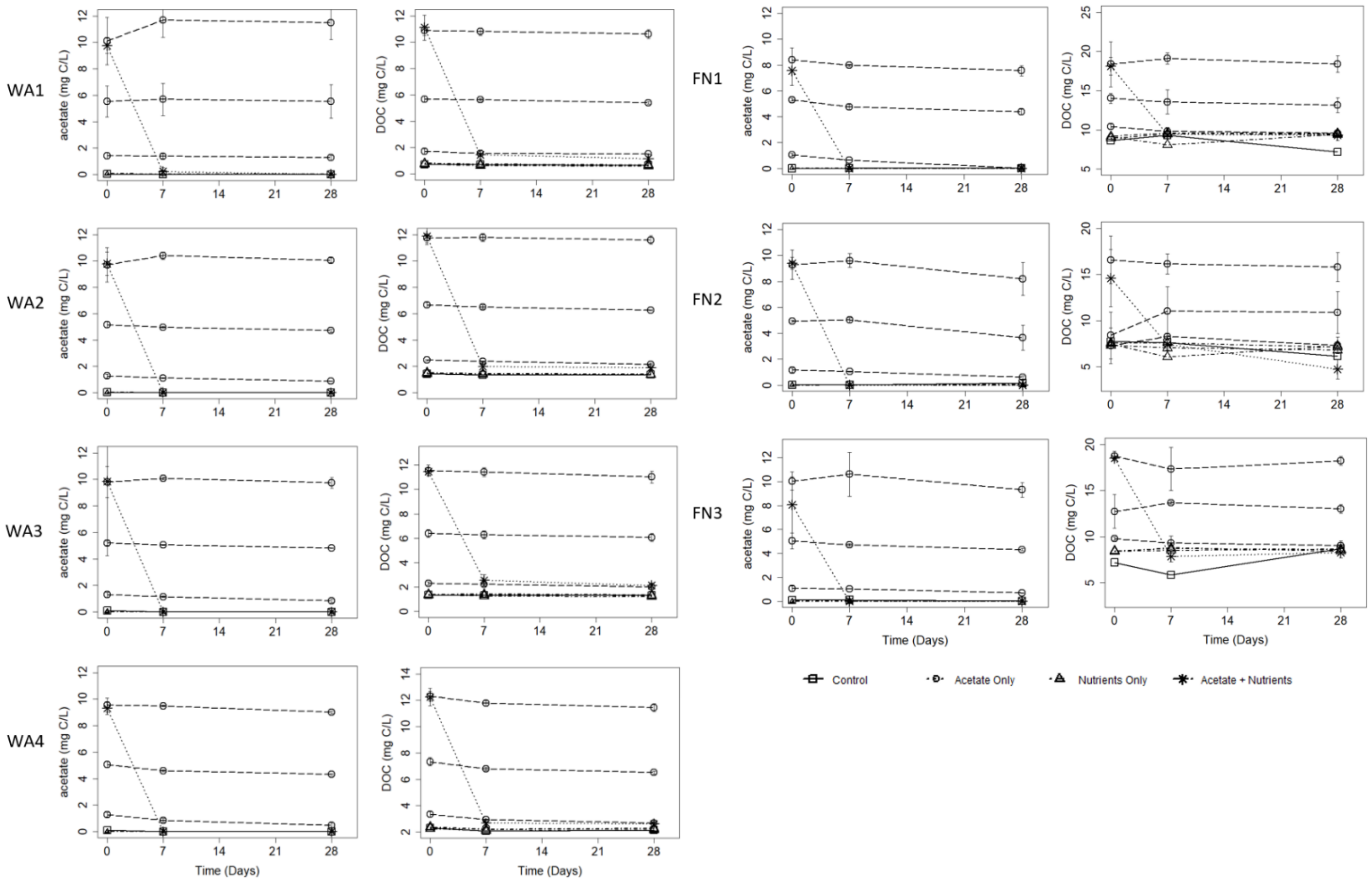

Figure S3: Example time series of acetate and DOC concentrations during incubations. Changes in acetate (left columns) and DOC (right columns) concentrations through time for the Western Alaska and Finland sites. Circles indicate analyte concentrations at sampling events (0, 7 and 28 days) and error bars show the standard deviation across the three replicates.

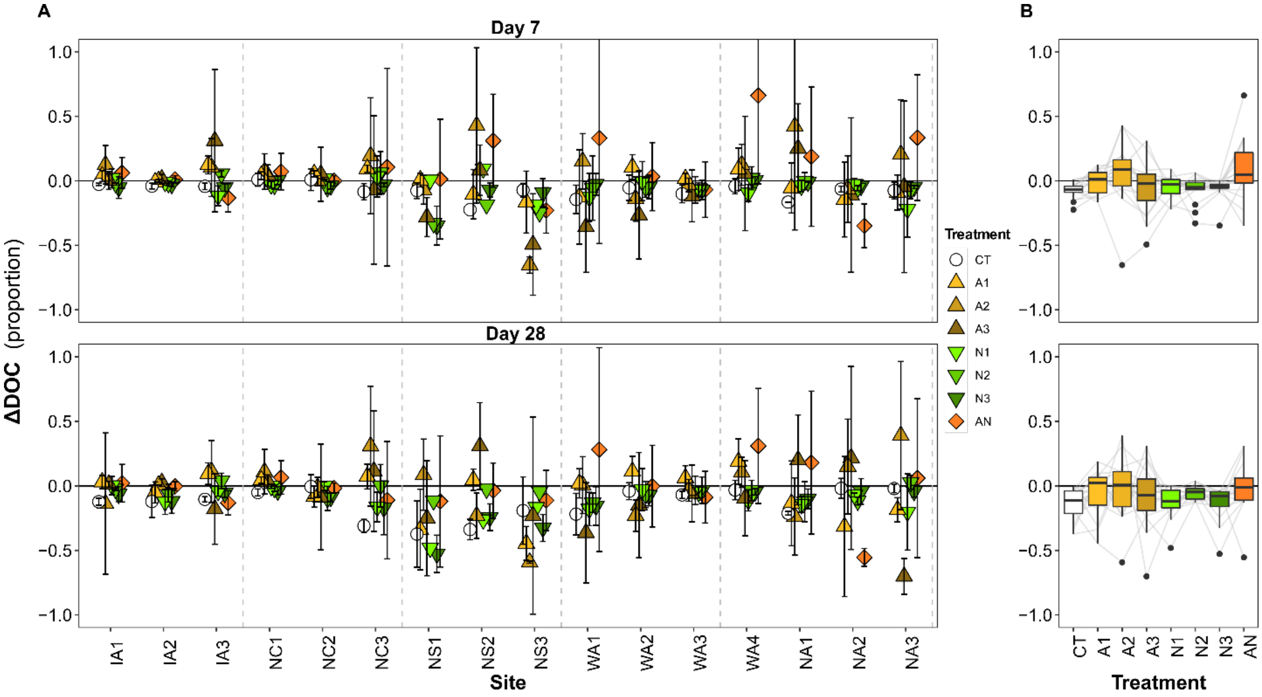

Figure S4: Proportional change in DOC concentration by site and treatment. A negative change represents a decrease in DOC concentration. Shapes represent the mean, and error bars show the standard deviation across the three replicates.

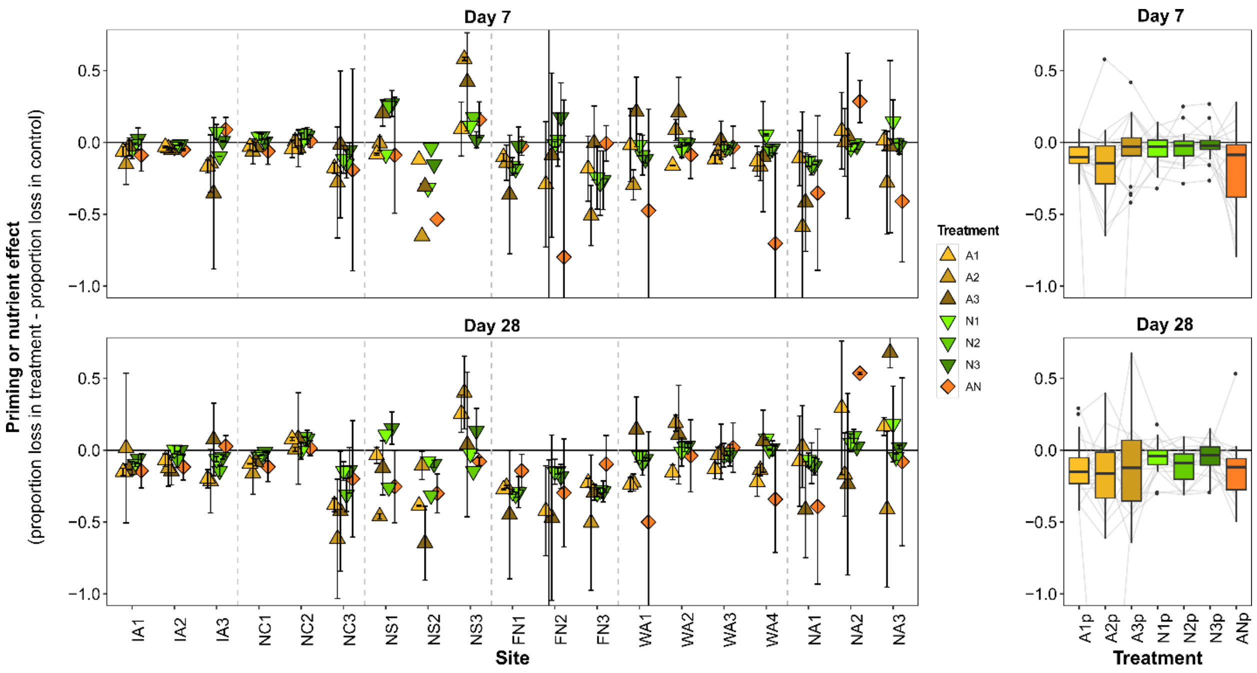

Figure S5: Priming and nutrient effects by site. Changes in acetate (left) and DOC (right) concentrations versus time for representative sites. Circles indicate analyte concentrations at sampling events (0, 7 and 28 days). Symbology follows Figure S4.

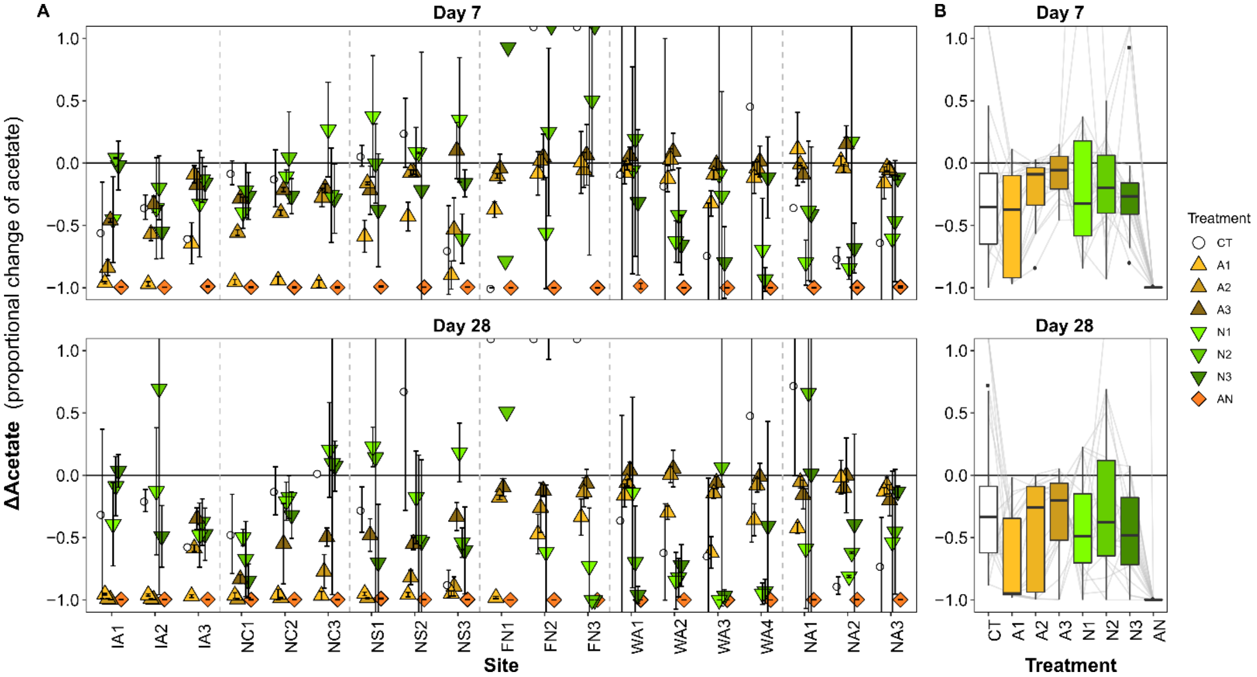

Figure S6: Change in acetate by site and treatment. Changes in acetate (left) and DOC (right) concentrations versus time for representative sites. Symbology follows Figure S4.

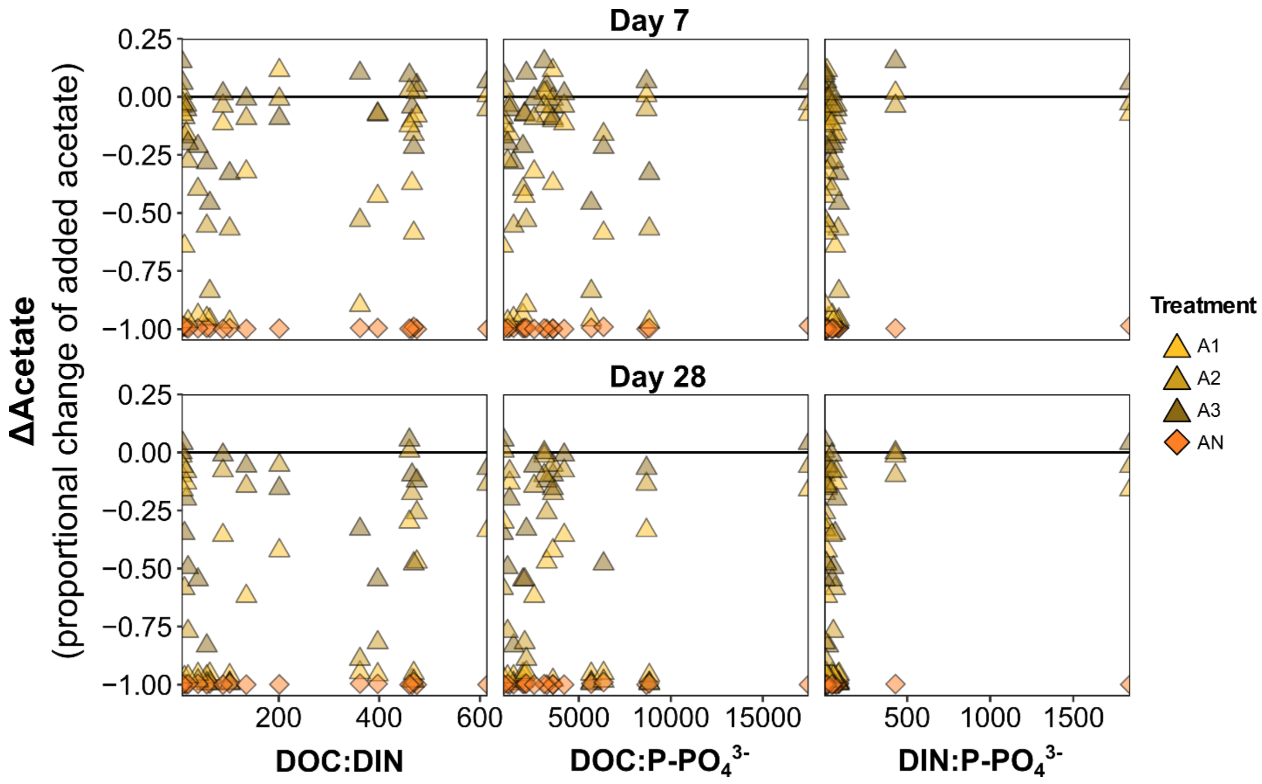

Figure S7: Correlations between change in added acetate and background stoichiometry. Ratios were calculated on a molar basis. There were no significant correlations at (α = 0.05) based on Spearman correlation analysis within treatment.

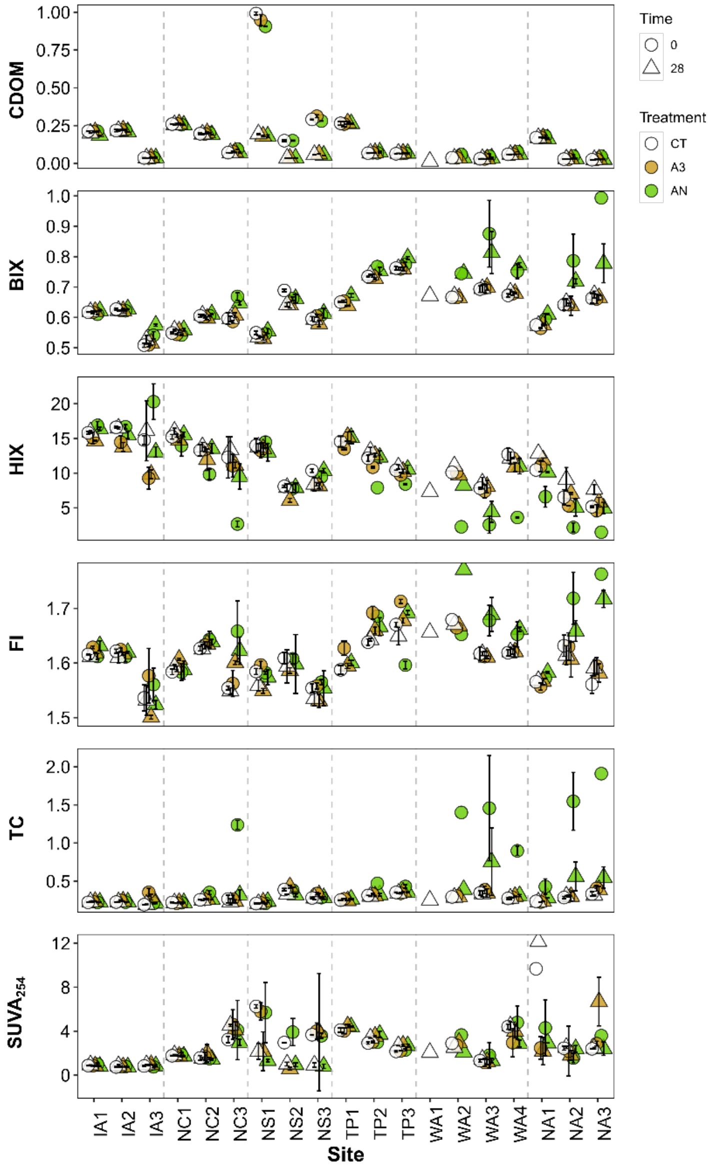

Figure S8: DOM optical properties at t_0 and t_{28}. Symbology follows Figure S4.

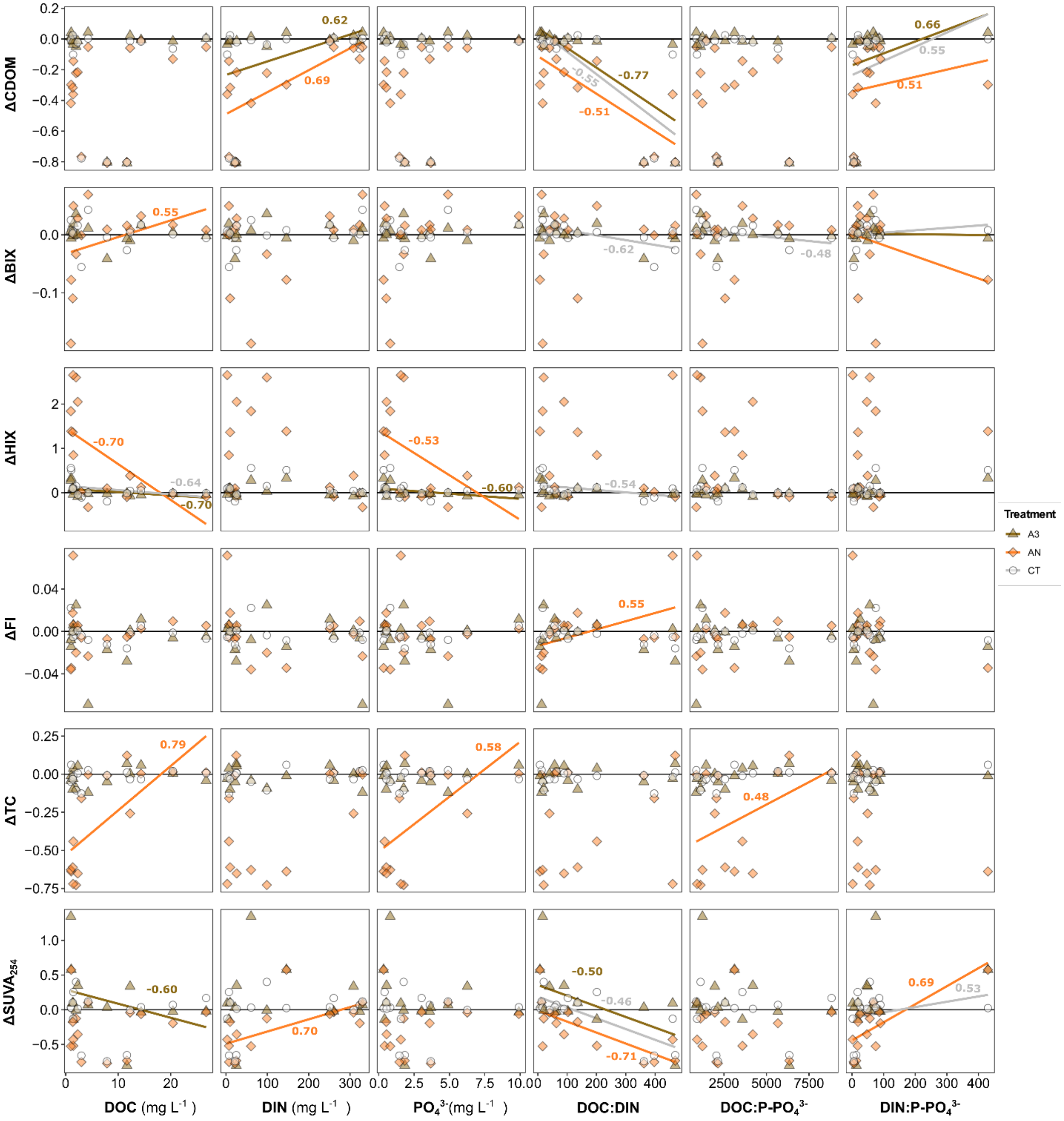

Figure S9: Relationships between change in optical properties and background chemistry. Spearman correlation coefficients for each treatment shown when significant ($p < 0.05$).

Detailed protocol used by each regional team

Could priming and nutrient effects from degrading permafrost alter dissolved organic matter dynamics in permafrost-zone waterways?

Participants (region code)

Arctic Alaska (AA): Ben Abbott, Jay Zarnetske, Gilles Pinay, Michelle Baker
Siberia Yedoma (SY): Joshua Dean, Jorien Vonk
Boreal Alaska (BA): Stephanie Ewing, Jon O'Donnell, Kim Wickland, Max Holmes
Tibetan Plateau (TP): Yuanhe Yang, Futing Liu
Scandinavia (SC): Paul Mann, Samuel Cottingham
Alaska permafrost (AP): Rob Spencer, Sadie Textor
Boreal Canada (BC): Suzanne Tank, Sarah Shakil, Scott Zolkos
Finland (FN): Pirkko Kortelainen, Jaana Kolehmainen

Background

It is unknown how hydrologic fluxes of organic matter will change in response to Arctic climate change, representing an important uncertainty in landscape carbon balance and habitat change in aquatic ecosystems (Laudon et al. 2012, Kicklighter et al. 2013, Abbott et al. 2016). Permafrost degradation is causing widespread release of biodegradable dissolved organic matter (BDOM) and inorganic nutrients (Vonk et al. 2013, Abbott et al. 2014, Ewing et al. 2015, Treat et al. 2016). While permafrost BDOM is rapidly mineralized or diluted in headwater streams, (Spencer et al. 2015, Mann et al. 2015, Drake et al. 2015), increased delivery of BDOM and nutrients to Arctic waterways could influence the turnover and mineralization of "modern" or background organic matter moving through freshwater and estuarine systems via priming and nutrient effects (Guenet et al. 2010, Rosemond et al. 2015). Because these lateral carbon fluxes are substantial (ca 120 tg yr^{-1} delivered to inland waters), priming of background DOM by permafrost BDOM or nutrients could influence landscape and continental-scale fluxes, but these interactions have not been quantified for Arctic or Boreal ecosystems (but see Dorado-García et al. 2015). This project will investigate the effects of BDOM and nutrients on DOM mineralization in headwater and mid-order streams. Sampling will occur during the growing season of 2016 in the following regions: Alaskan Tundra and Boreal Forest, Tibetan Plateau, Siberian Yedoma region, and Scandinavian boreal forest.

Priming and nutrient effects will be quantified by measuring DOC drawdown during incubations (Abbott et al. 2014, Vonk et al. 2015) with and without biodegradable carbon (acetate) and nutrients. Sites will span a range of stream orders allowing us to quantify priming and nutrient effects in headwaters where permafrost effects will likely be greatest relative to background DOM fluxes, and in larger streams where currently the permafrost signal is washed out. Separation of added and ambient DOM losses will be determined by Bayesian inverse modelling with initial coefficients of decay constrained by incubations of the substrates (Hotchkiss et al. 2014), confirmed with direct quantification of acetate by ion chromatography. We will use optical properties (e.g. EEMs, CDOM, SUVA) and FTICR-MS sampling at the beginning and end of the incubation to investigate how DOM optical properties and composition determine initial biodegradability and what compounds were preferentially consumed. The study will be the first quantification of the priming effect in Arctic freshwater ecosystems and will

address the conflicting evidence of the relationship between nutrient availability and DOM decomposition (Holmes et al. 2008, Wickland et al. 2012, Abbott et al. 2014).

Protocol

- **Experimental design**
  - We will quantify nutrient and priming effects moving from headwaters to larger rivers. For each region, samples should be collected from three nested catchments (including small headwater streams to larger rivers) to see how sensitive background or modern DOM is to priming and nutrient effects, and if this sensitivity changes during transport from land to sea.

- **Sample collection**
  - At each location, water should be filtered to 0.7 µm (GF/F) the same day as sampling and incubations should be set up within one to two days if possible.
    - From each site collect:
      - 6 L of 0.7 µm-filtered water for setting up incubations.
      - Three 30 mL samples of 0.2 µm-filtered water in amber vials for EEMs analysis in the Baker lab (refrigerated upon return to lab).
      - Three 60 mL of 0.2 µm-filtered water for background chemistry analyses (frozen upon return to lab).
      - Standard water chemistry parameters (estimate of discharge, conductivity, pH, O2 etc) and catchment characteristics (catchment size, permafrost extent, vegetation cover, wetland/peatland extent, basic climatological parameters, etc).

- **Incubation setup**
  - Incubations are run in 250 ml glass bottles at room temperature (20C). Remember that cleanliness is key throughout the experiment (ideally acid wash, ash, and rinse everything that can be prior to use) and avoid substances that can leach carbon (e.g. rubber caps).
  - At the beginning of the incubation, pour 200 mL of 0.7µm-filtered water into each glass bottle (filtering to 0.7µm removes particulates but lets enough microorganisms through to assure decomposition).
  - There are eight treatments run in triplicate for each site, two bottles for FTICR-MS from one site, and 5 blanks (bottles filled with DI) for each region (79 incubation bottles for each set of 3 sites).
  - Target acetate and nutrient concentrations are based on observed concentrations in thermokarst outflows (the likely upper limit of what soil water and headwater stream DOM would be exposed to). You will add one mL from the relevant stock solution (preparation described at end of protocol) for each treatment.
    - Control (nothing added)
    - 1, 5, and 10 mg/L of added acetate (CH_3COONa)
    - Low, medium, and high N/P treatments ($NaNO_3$, NH_4Cl, K_2HPO_4)

        - 10 mg/L of Acetate + high N/P (1 mL from the high acetate and nutrient treatments)

*Remember to pour off and freeze subsamples of each of your stock solutions and DI water into 15 mL Falcon tubes to be sent to Stephanie for analysis. This means 7 additional tubes.

    - Because ~60 mL at the initial and final sampling are needed for FTICR-MS analysis (120 mL total), set up an additional "control" and "AN" (10 mg/L acetate and high N/P) bottle preferably for the headwater site.
    - Remember to open and swirl (agitate gently) all the bottles of the incubation once a week to ensure adequate oxygen.

- **Incubation samplings**
    - Sample the incubations at 0, 7, and 28 days (216 DOC/acetate measurements per region or 1080 samples in all).
        - DOC and acetate sampling for all treatments consists of pouring off 14 mL of sample (no filtering needed) into 15 mL freezable Falcon tubes, and freezing. *To minimize cracking, do not fill tubes completely and put them in a horizontal or slanted position (do not put them upright in styrofoam racks during freezing). At the end of the experiment, all frozen samples should be shipped on dry ice to Stephanie Ewing.
        - For the initial and final samplings of the control, high acetate, and nutrient+acetate treatments, filter an additional 30 mL of sample water to 0.2 μm into an amber vial for EEMs analysis and comparison of mineralization and microbial uptake (difference in DOC concentration between 0.2 and 0.7μm-filtered water). *These samples must not be frozen. They should be stored in the refrigerator until the end of the experiment and then sent to Michelle Baker.
        - At the initial and final sampling of the FTICR-MS incubations, filter with an ashed GF/F 50 mL of water into a 60 mL pre-leached polycarbonate or HDPE bottle (10% HCl for 72 hrs and triple rinsed with Milli-Q and then rinsed with a little sample) and frozen immediately. Wait until Rob Spencer sends out a message with the shipping info and then send to him.
    - Labelling convention
        - See "Participants" section above for your group's region code (e.g. AA for Arctic Alaska).
        - Treatments should be labelled as follows:
            - Control: Ct
            - Acetate 1 mg: A1
            - Acetate 5 mg: A2
            - Acetate 10 mg: A3
            - Nutrient low: N1
            - Nutrient medium: N2
            - Nutrient high: N3
            - Acetate+N: AN
            - Blank: Bk

- Each vial (for EEMs), tube (for DOC/acetate), or bottle (for FTICR-MS) should be labeled with the region, a number for the site, a letter for the treatment, a number for the replicate, and a number for the sampling,. For example, the second replicate of the initial sampling of the 5 mg acetate treatment from the Arctic Alaska group's site 1 will be labelled *AA.1.A2. R2.T0*

- **Sample processing and shipping**
  - Ship acetate and DOC samples (frozen 15 mL Falcon tubes), background nutrient samples (frozen 60 mL HDPE bottles), and treatment stock solutions and DI water (frozen 15 mL Falcon tubes) with ice packs at the end of the incubations to:
    Stephanie A. Ewing
    PO Box 173120
    Dept. of Land Resources & Environmental Sciences
    Montana State University
    Bozeman, MT 59717

  - Ship EEMs samples (unfrozen 40 mL amber vials) in an insulated shipping box or cooler at the end of the incubations to:
    Michelle Baker
    5305 Old Main Hill
    Department of Biology
    Utah State University
    Logan, UT 84322 USA

  - Ship FTICR samples (frozen 60 mL HDPE bottles) with ice packs after confirming with Sadie:
    Sadie Textor
    Dept. Earth, Ocean & Atmospheric Science
    Oceanography / Statistics Building
    Florida State University
    Tallahassee
    FL 32306

Supply list

1. Glass incubation bottles of 200mL capacity or greater (24 per site plus 2 for FTICR and 5 for blanks; e.g. link here)
2. Glass fiber filters of 0.7µm effective pore size (enough to filter 6 L of water from each site; e.g. Whatman GF/F)
3. 0.22 µm polyethersulfone membrane filters for EEMs samples (18 per site; e.g. link here or Millipore Millex PES capsules)
4. 40 mL amber storage vials for EEMs samples (18 per site plus the 9 background samples that were collected during initial sampling; e.g. SKU:276840)
5. 15 mL freezable Falcon tubes for DOC and acetate analysis (180 per site; e.g. SKU:)
6. 60 mL HDPE bottles for background water chemistry (3 per site; e.g. SKU:)

7. Reagents for nutrient and acetate treatments
    a. Sodium acetate anhydrous (CH_3COONa--also called C2H3NaO2)
    b. Sodium nitrate ($NaNO_3$)
    c. Ammonium chloride (NH_4Cl)
    d. Dipotassium phosphate (K_2HPO_4)
8. General lab supplies including
    a. 100 mL volumetric flasks (x3)
    b. 250 mL volumetric flasks (x3)
    c. Pipettor accurate for 1 mL
    d. Syringes for filtering

Preparation of treatment stock solutions

- 1 mg acetate treatment (100 mL volumetric flask)
    - 70 mg C2H2NaO2
- 5 mg acetate treatment (100 mL volumetric flask)
    - 350 mg C2H2NaO2
- 10 mg acetate treatment (100 mL volumetric flask)
    - 700 mg C2H2NaO2
- Low nutrient treatment (250 mL volumetric flask)
    - 100 mg NH4Cl
    - 32 mg $NaNO_3$
    - 6 mg K2HPO4
- Medium nutrient treatment (250 mL volumetric flask)
    - 500 mg NH4Cl
    - 155 mg $NaNO_3$
    - 30 mg K2HPO4
- High nutrient treatment (250 mL volumetric flask)
    - 1000 mg NH4Cl
    - 320 mg $NaNO_3$
    - 60 mg K2HPO4

*Remember to pour off and freeze subsamples of each of your stock solutions and DI water into 15 mL Falcon tubes to be sent to Stephanie for analysis. This means 7 additional tubes.

Abbreviated protocol

1. Get water in field
    a. 6L for incubation
    b. Three 60 mL bottles filtered to 0.2 for "background" water chemistry (freeze)
    c. Three 40 mL amber vials filtered to 0.2 for EEMs analysis (refrigerate)
2. Set up incubation
    a. Filter the 6 L with 0.7 um glass fiber filter
    b. Pour 200 mL of water into 250 mL incubation bottles (for each site there should be 24 bottles: 8 treatments x 3 replicates). Additionally fill up 2 extra bottles for FTICR-MS

(one to be run as a control and the other as a high acetate+nutrients treatment "AN") from your most interesting site (preferably the headwater site) and set up 5 blanks with DI water.

c. Add 1 mL of treatment stock solution as appropriate (2 mL for acetate+nutrients treatment—one for each A and N)

3. Samplings
    a. At 0, 7, and 28 days sample all the treatments (including blanks)
        i. Unfiltered water into 15 mL falcon tubes (3 tubes for acetate treatments, 2 tubes for all others). Freeze.
    b. At 0 and 28 days additionally sample
        i. Control and acetate+nutrients treatment FTICR-MS bottles (50 mL of 0.7 um GF/F filtered water into leached HDPE bottles). Freeze.
        ii. Control, high acetate, and acetate+nutrients treatments for EEMs analysis (30 mL of 0.2 um filtered water into EEMs vial). Refrigerate.
4. Send samples
    a. DOC/acetate and nutrients to Stephanie (frozen with dry ice)
    b. EEMs to Michelle (refrigerated)
    c. FTICR to Rob (frozen with dry ice) *only send these samples when Rob lets us know.
5. Publish paper
    a. Analyze data
    b. Make cool figures
    c. Write some snappy and insightful text
    d. Publish in PNAS, Nature, or Science

References

Abbott, B. W., J. B. Jones, E. A. G. Schuur, F. S. C. III, W. B. Bowden, M. S. Bret-Harte, H. E. Epstein, M. D. Flannigan, T. K. Harms, T. N. Hollingsworth, M. C. Mack, A. D. McGuire, S. M. Natali, A. V. Rocha, S. E. Tank, M. R. Turetsky, J. E. Vonk, K. P. Wickland, G. R. Aiken, H. D. Alexander, R. M. W. Amon, B. W. Benscoter, Yves Bergeron, K. Bishop, O. Blarquez, B. Bond-Lamberty, A. L. Breen, I. Buffam, Yihua Cai, C. Carcaillet, S. K. Carey, J. M. Chen, H. Y. H. Chen, T. R. Christensen, L. W. Cooper, J. H. C. Cornelissen, W. J. de Groot, T. H. DeLuca, E. Dorrepaal, N. Fetcher, J. C. Finlay, B. C. Forbes, N. H. F. French, S. Gauthier, M. P. Girardin, S. J. Goetz, J. G. Goldammer, L. Gough, P. Grogan, L. Guo, P. E. Higuera, L. Hinzman, F. S. Hu, Gustaf Hugelius, E. E. Jafarov, R. Jandt, J. F. Johnstone, J. Karlsson, E. S. Kasischke, Gerhard Kattner, R. Kelly, F. Keuper, G. W. Kling, P. Kortelainen, J. Kouki, P. Kuhry, Hjalmar Laudon, I. Laurion, R. W. Macdonald, P. J. Mann, P. J. Martikainen, J. W. McClelland, Ulf Molau, S. F. Oberbauer, D. Olefeldt, D. Paré, M.-A. Parisien, S. Payette, Changhui Peng, O. S. Pokrovsky, E. B. Rastetter, P. A. Raymond, M. K. Raynolds, G. Rein, J. F. Reynolds, M. Robard, B. M. Rogers, C. Schädel, K. Schaefer, I. K. Schmidt, Anatoly Shvidenko, J. Sky, R. G. M. Spencer, G. Starr, R. G. Striegl, R. Teisserenc, L. J. Tranvik, T. Virtanen, J. M. Welker, and S. Zimov. 2016. Biomass offsets little or none of permafrost carbon release from soils, streams, and wildfire: an expert assessment. Environmental Research Letters 11:34014.

Abbott, B. W., J. R. Larouche, J. B. Jones, W. B. Bowden, and A. W. Balser. 2014. Elevated dissolved organic carbon biodegradability from thawing and collapsing permafrost:

Permafrost carbon biodegradability. Journal of Geophysical Research: Biogeosciences 119:2049–2063.
Dorado-García, I., J. Syväranta, S. P. Devlin, J. M. Medina-Sánchez, and R. I. Jones. 2015. Experimental assessment of a possible microbial priming effect in a humic boreal lake. Aquatic Sciences 78:191–202.
Drake, T. W., K. P. Wickland, R. G. M. Spencer, D. M. McKnight, and R. G. Striegl. 2015. Ancient low–molecular-weight organic acids in permafrost fuel rapid carbon dioxide production upon thaw. Proceedings of the National Academy of Sciences 112:13946–13951.
Ewing, S. A., J. A. O'Donnell, G. R. Aiken, K. Butler, D. Butman, L. Windham-Myers, and M. Z. Kanevskiy. 2015. Labile C and N in Pleistocene permafrost: Long-term anoxia may drive the magnitude of DOC loss upon permafrost thaw. Geophysical Research Letters:2015GL066296.
Guenet, B., M. Danger, L. Abbadie, and G. Lacroix. 2010. Priming effect: bridging the gap between terrestrial and aquatic ecology. Ecology 91:2850–2861.
Holmes, R. M., J. W. McClelland, P. A. Raymond, B. B. Frazer, B. J. Peterson, and M. Stieglitz. 2008. Lability of DOC transported by Alaskan rivers to the Arctic Ocean. Geophysical Research Letters 35:L03402.
Hotchkiss, E. R., R. O. Hall, M. A. Baker, E. J. Rosi-Marshall, and J. L. Tank. 2014. Modeling priming effects on microbial consumption of dissolved organic carbon in rivers. Journal of Geophysical Research: Biogeosciences 119:982–995.
Kicklighter, D. W., D. J. Hayes, J. W. McClelland, B. J. Peterson, A. D. McGuire, and J. M. Melillo. 2013. Insights and issues with simulating terrestrial DOC loading of Arctic river networks. Ecological Applications 23:1817–1836.
Laudon, H., J. Buttle, S. K. Carey, J. McDonnell, K. McGuire, J. Seibert, J. Shanley, C. Soulsby, and D. Tetzlaff. 2012. Cross-regional prediction of long-term trajectory of stream water DOC response to climate change: CROSS-REGIONAL PREDICTION OF DOC. Geophysical Research Letters 39:n/a-n/a.
Mann, P. J., T. I. Eglinton, C. P. McIntyre, N. Zimov, A. Davydova, J. E. Vonk, R. M. Holmes, and R. G. M. Spencer. 2015. Utilization of ancient permafrost carbon in headwaters of Arctic fluvial networks. Nature Communications 6:7856.
Rosemond, A. D., J. P. Benstead, P. M. Bumpers, V. Gulis, J. S. Kominoski, D. W. P. Manning, K. Suberkropp, and J. B. Wallace. 2015. Experimental nutrient additions accelerate terrestrial carbon loss from stream ecosystems. Science 347:1142–1145.
Spencer, R. G. M., P. J. Mann, T. Dittmar, T. I. Eglinton, C. McIntyre, R. M. Holmes, N. Zimov, and A. Stubbins. 2015. Detecting the signature of permafrost thaw in Arctic rivers. Geophysical Research Letters 42:2830–2835.
Treat, C. C., W. M. Wollheim, R. K. Varner, and W. B. Bowden. 2016. Longer thaw seasons increase nitrogen availability for leaching during fall in tundra soils. Environmental Research Letters 11:64013.
Vonk, J. E., P. J. Mann, S. Davydov, A. Davydova, R. G. M. Spencer, J. Schade, W. V. Sobczak, N. Zimov, S. Zimov, E. Bulygina, T. I. Eglinton, and R. M. Holmes. 2013. High biolability of ancient permafrost carbon upon thaw. Geophysical Research Letters 40:2689–2693.
Vonk, J. E., S. E. Tank, P. J. Mann, R. G. M. Spencer, C. C. Treat, R. G. Striegl, B. W. Abbott, and K. P. Wickland. 2015. Biodegradability of dissolved organic carbon in permafrost soils and aquatic systems: a meta-analysis. Biogeosciences 12:6915–6930.

Wickland, K. P., G. R. Aiken, K. Butler, M. M. Dornblaser, R. G. M. Spencer, and R. G. Striegl. 2012. Biodegradability of dissolved organic carbon in the Yukon River and its tributaries: Seasonality and importance of inorganic nitrogen. Global Biogeochemical Cycles 26:GB0E03.
